# Lysosomal acidification impairment in astrocyte-mediated neuroinflammation

**DOI:** 10.1186/s12974-025-03410-w

**Published:** 2025-03-10

**Authors:** Jialiu Zeng, Jonathan Indajang, David Pitt, Chih Hung Lo

**Affiliations:** 1 Department of Biomedical and Chemical Engineering, Syracuse University, Syracuse, NY 13244 USA; 2 Interdisciplinary Neuroscience Program, Syracuse University, Syracuse, NY 13244 USA; 3 Meinig School of Biomedical Engineering, Cornell University, Ithaca, NY 14853 USA; 4 Department of Neurology, Yale School of Medicine, New Haven, CT 06511 USA; 5 Department of Biology, Syracuse University, Syracuse, NY 13244 USA

**Keywords:** Lysosomal acidification, Lysosomal alkalization, Autophagy, Phagocytosis, Metabolic dysfunction, Acidic nanoparticles, Glial crosstalk, Neurodegeneration, Neuroinflammation, Neuroprotective

## Abstract

Astrocytes are a major cell type in the central nervous system (CNS) that play a key role in regulating homeostatic functions, responding to injuries, and maintaining the blood-brain barrier. Astrocytes also regulate neuronal functions and survival by modulating myelination and degradation of pathological toxic protein aggregates. Astrocytes have recently been proposed to possess both autophagic activity and active phagocytic capability which largely depend on sufficiently acidified lysosomes for complete degradation of cellular cargos. Defective lysosomal acidification in astrocytes impairs their autophagic and phagocytic functions, resulting in the accumulation of cellular debris, excessive myelin and lipids, and toxic protein aggregates, which ultimately contributes to the propagation of neuroinflammation and neurodegenerative pathology. Restoration of lysosomal acidification in impaired astrocytes represent new neuroprotective strategy and therapeutic direction. In this review, we summarize pathogenic factors, including neuroinflammatory signaling, metabolic stressors, myelin and lipid mediated toxicity, and toxic protein aggregates, that contribute to lysosomal acidification impairment and associated autophagic and phagocytic dysfunction in astrocytes. We discuss the role of lysosomal acidification dysfunction in astrocyte-mediated neuroinflammation primarily in the context of neurodegenerative diseases along with other brain injuries. We then highlight re-acidification of impaired lysosomes as a therapeutic strategy to restore autophagic and phagocytic functions as well as lysosomal degradative capacity in astrocytes. We conclude by providing future perspectives on the role of astrocytes as phagocytes and their crosstalk with other CNS cells to impart neurodegenerative or neuroprotective effects.

## Introduction

**Fig. 1:**
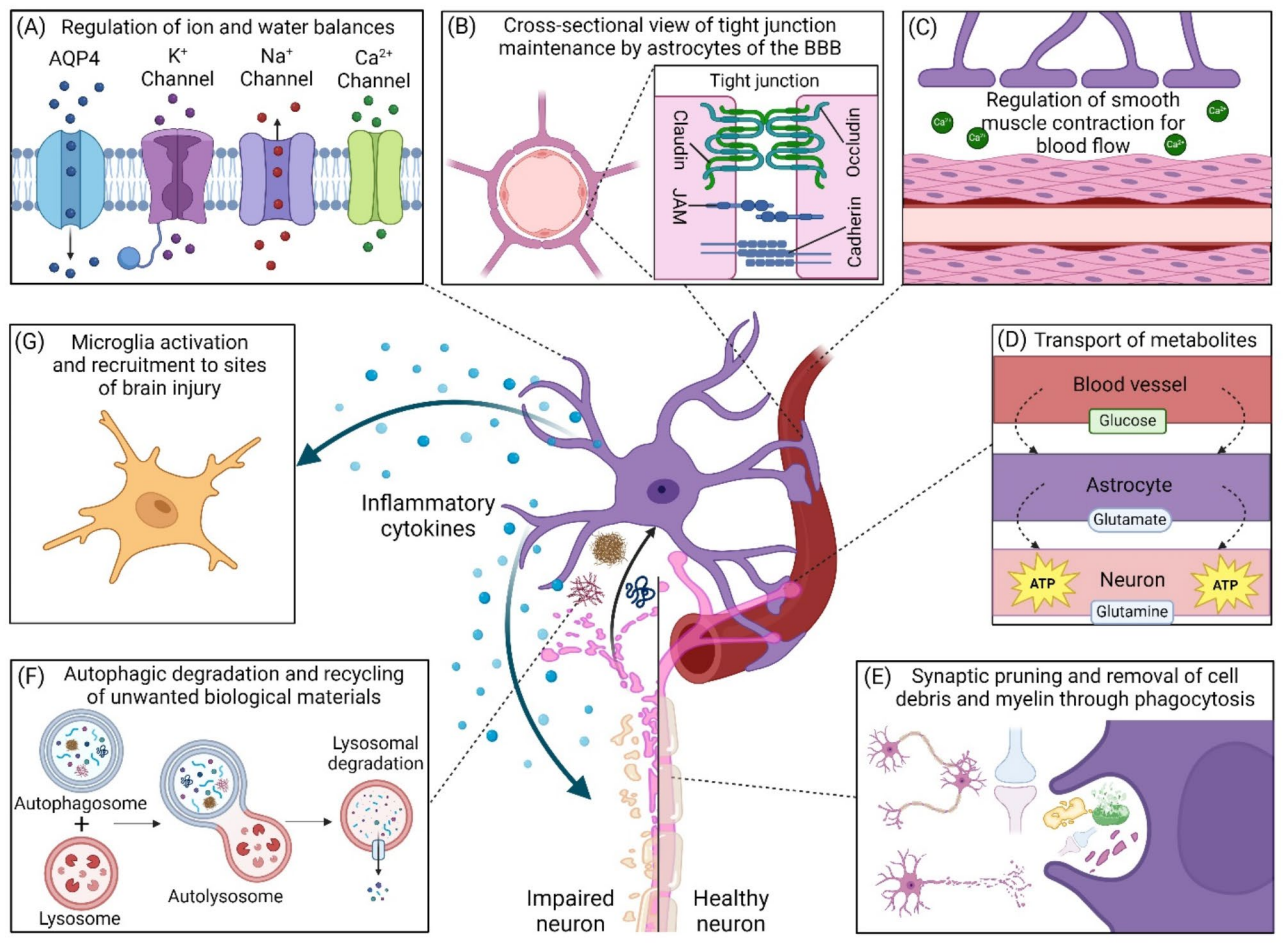
The multifaceted roles of astrocytes in the brain. (**A**) Astrocytes control ion homeostasis and water exchange in the brain microenvironment by regulating channel proteins including aquaporin-4 (AQP4) water channel as well as potassium, sodium, and calcium channels. (**B**) Astrocytes support the formation of tight junctions (e.g., claudin, occludin, junctional adhesion molecules (JAM), and cadherin) and the maintenance of epithelial cells at the blood-brain barrier (BBB). (**C**) Contraction and blood flow movement of the BBB is mediated by smooth muscle tissue that respond to Ca^2+^ ions released and regulated by astrocytes. (**D**) Astrocytes mediate nutrient transport to neurons to regulate neuronal metabolism. (**E**) Astrocytes operate as phagocytes to carry out synaptic pruning as well as remove cell debris, damaged organelles, and myelin. (**F**) Astrocytes participate in autophagic degradation of intracellular toxic protein aggregates, myelin/lipids and cellular debris phagocytosed from damaged neurons. (**G**) Astrocytes release inflammatory cytokines that can recruit microglia to sites of brain injury and/or induce neuronal impairment and death. The figure was created with BioRender.com

Astrocytes are a major class of glial cells found in the central nervous system (CNS), representing 19 to 40% of the glial population [1]. Under normal condition, astrocytes are responsible for maintaining homeostasis including regulation of ion and water balance [2], maintenance of the blood-brain barrier (BBB) [3], and regulation of local cerebral blood flow [2, 3] (Fig. 1A-C). Astrocytes also help to provide support for neuronal metabolic functions [4] and maintain synaptic homeostasis through modulating synaptic formation, maturation, and elimination [5, 6] (Fig. 1D-E). Furthermore, astrocytes play key roles in phagocytic uptake of cellular debris, myelin/lipids, and toxic protein aggregates [7, 8] as well as modulation of neuroimmune responses [9] (Fig. 1F-G). Upon exposure to external stimuli, astrocytes become reactive and exhibit cellular heterogeneity, including alterations in cell morphology and functions, gene expression profiles, cytokine production levels, as well as their response to injuries, which have implications towards brain inflammation and neuronal death [10–14].

**Fig. 2:**
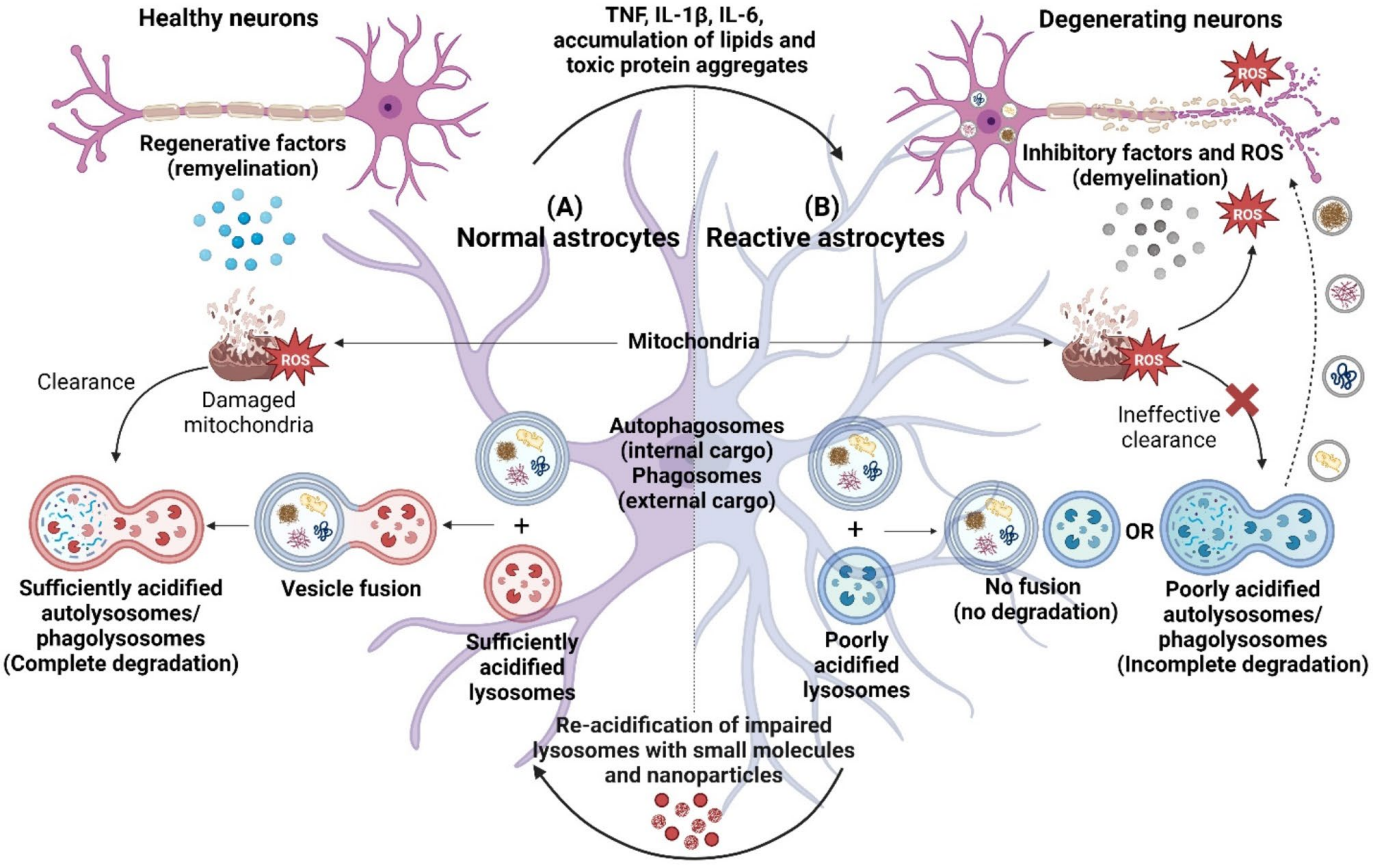
Autophagic and phagocytic degradative functions in normal and reactive astrocytes. (**A**) In normal astrocytes, lysosomes maintain a sufficiently acidic environment, enabling proper vesicle fusion and optimal autophagic/phagocytic activities, including the clearance of damaged mitochondria and myelin debris, thereby maintaining neuronal health. In addition, astrocytes release regenerative factors which contribute to neuron remyelination. (**B**) In reactive astrocytes under exposure to pro-inflammatory cytokines, excessive lipids, and toxic protein aggregates, lysosomal acidification is impaired (poorly acidified lysosomes), leading to inhibition of autophagic/phagocytic activities. As a result, there is reduced mitochondrial turnover and increased accumulation of damaged mitochondria, as well as release of neurotoxic factors such as ROS. In addition, damaged astrocytes can release undegraded toxic materials as well as inhibitory factors that further impair neuronal function. Re-acidification of impaired lysosomes by lysosome-targeting small molecules and nanoparticles restores autophagic/phagocytic functions in astrocytes, allowing for effective clearance of neurotoxic factors to maintain neuronal health. The figure was created with BioRender.com

Recent studies have highlighted that autophagic and phagocytic processes play key regulatory roles in astrocytic degradation capability and impairments in these functions could contribute to neuroinflammation and neurodegeneration [15–17]. In addition to the processing of external cargo by phagocytosis [18], the processing of internal cargo by autophagy is important in astrocyte differentiation and maturation as well as regulation of mitochondrial dynamics, reactive oxygen species (ROS) generation, neuroimmune response, and cell death [19]. In astrocyte autophagy and phagocytosis, fusion of autophagosomes and phagosomes with sufficiently acidified lysosomes as maintained by the lysosomal vacuolar (H+)-ATPase (V-ATPase) is essential for their degradative functions [20]. In the homeostatic state, astrocyte autophagy and phagocytosis are functional in the presence of optimal lysosomal acidification and these processes maintain cellular homeostasis, support axonal health, and regulate myelination, contributing to neuronal plasticity, functions, and survival [21, 22] (Fig. 2A).

In their reactive states, it has been demonstrated that an elevation of lysosomal pH or defective lysosomal acidification decreases the efficiency and effectiveness of astrocytes to perform autophagic and phagocytic functions [23, 24]. The resulting accumulation of damaged organelles, myelin debris, and toxic protein aggregates as well as the release of inhibitory factors and ROS further propagate neuroinflammation and drive neurodegeneration [25, 26] (Fig. 2B). Under aging or diseased conditions, there are evidence of synaptic and autophagosomal proteins as well as toxic protein aggregates accumulated and colocalized with poorly acidified lysosomes in reactive astrocytes [27–30]. However, the role of different stimuli and their molecular mechanisms associated with lysosomal acidification impairment in astrocytes are unclear and remain to be clarified. While the accumulation of unwanted and toxic materials may be a consequence of lysosomal dysfunction, it is important to note that these materials could also be the initial triggers in impairing lysosomal acidification.

**Fig. 3:**
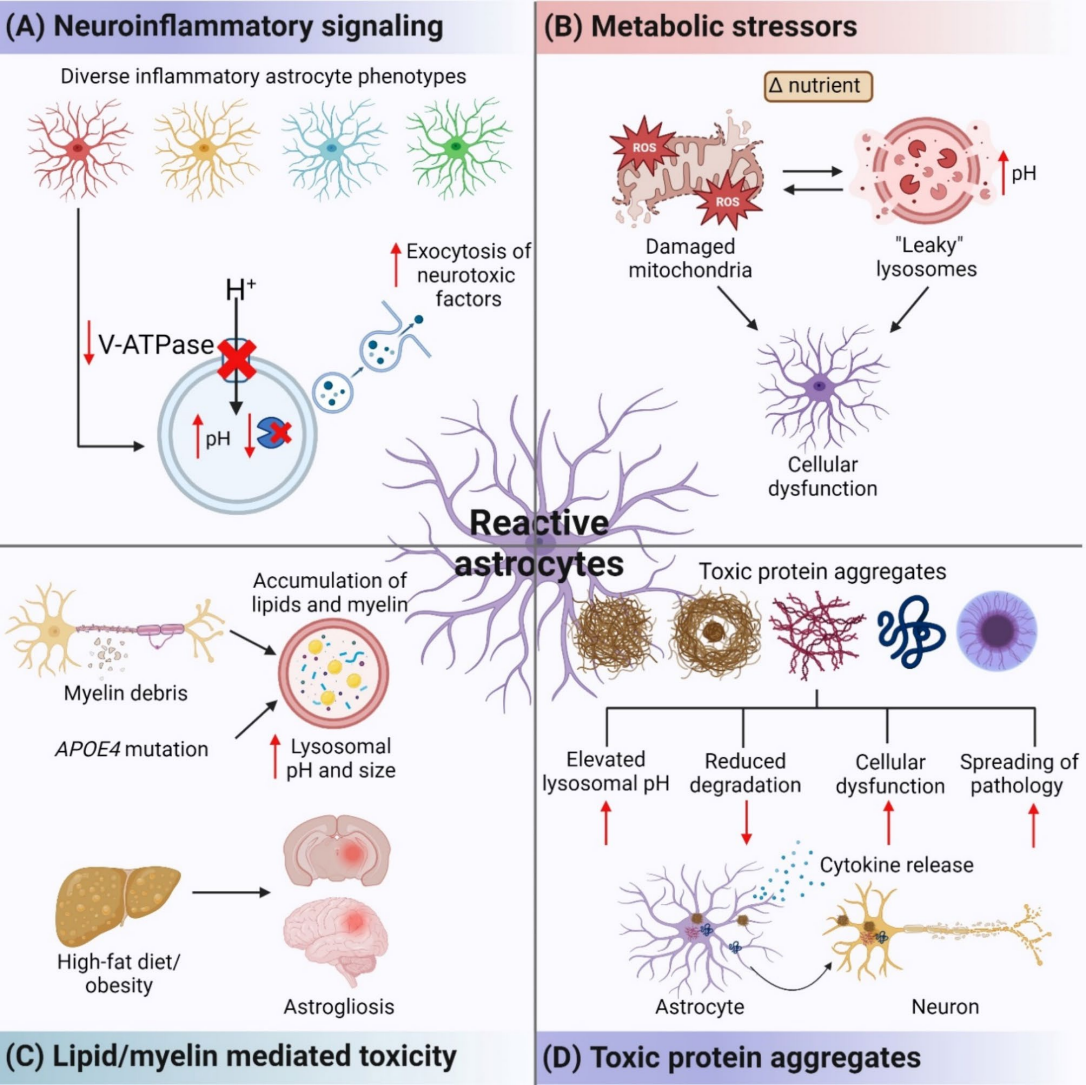
Factors affecting lysosomal acidification dysfunction in astrocytes. (**A**) Exposure to different cytokines triggers neuroinflammatory signaling that induce diverse astrocyte inflammatory phenotypes. Cytokines exposure leads to a reduction of lysosomal V-ATPase levels, leading to elevated lysosomal pH and reduced lysosomal enzyme degradative capacity. Chronic autolysosomal buildup due to incomplete degradation leads to exocytosis of neurotoxic factors which impair surrounding neurons. (**B**) Changes in nutrient levels induce metabolic stress which lead to mitochondrial dysfunction and impaired lysosomal acidification. (**C**) In astrocytes with lipids or myelin accumulation, lysosomal size is increased along with elevated lysosomal pH. In addition, high fat diet intake and metabolic disorders that affect the peripheral organs can also affect astrocyte function and reactivity. (**D**) Toxic proteins aggregates taken up by astrocytes localized into lysosomes and impaired lysosomal acidification, resulting in cellular dysfunction and spreading of pathology due to inefficient degradation and increased release of the toxic materials. The figure was created with BioRender.com

In this review, we summarize the role of neuroinflammatory signaling, metabolic stressors, myelin and lipid mediated toxicity, and toxic protein aggregates in lysosomal acidification impairment and associated autophagic and phagocytic dysfunction in astrocytes (Fig. 3). We discuss these pathogenic factors primarily in the context of neurodegenerative diseases along with other brain injuries that provide insights to the role of lysosomal acidification dysfunction in astrocyte-mediated neuroinflammation. We provide some insight into the feedback mechanisms between pathogenic factors and lysosomal dysregulation, both regarding how astrocytic dysregulation is initiated by pathogenic factors, and how eventual dysregulation accelerates neural degeneration. We then highlight current therapeutic strategies to re-acidify lysosomes and restore astrocyte autophagic and phagocytic functions. We conclude by providing future perspectives on the role of astrocytes as phagocytes and their cooperative role with other CNS cells such as microglia and neurons to mediate neurodegeneration and neuroprotection.

## Neuroinflammatory signaling

Inflammatory reactive astrocytes with reduced homeostatic functions can be neurotoxic, contributing to neurodegenerative diseases including Alzheimer’s disease (AD), Parkinson’s disease (PD), and multiple sclerosis (MS). Reactive astrocytes subjected to microglia-derived pro-inflammatory cytokines such as a combination of tumor necrosis factor (TNF), interleukin-1α (IL-1α), and complement component 1q (C1q) have reduced ability to promote neuronal survival, synaptogenesis and phagocytosis, and can induce neuronal death in human AD, PD and MS tissues [31]. Different cytokine cocktails may induce different astrocyte phenotypes which could be neurotoxic or neuroprotective [31–33]. Defective lysosomal acidification has been shown to be associated with neurotoxic reactive astrocytes, age-related inflammation, and consequentially contribute to neurodegenerative diseases [33, 34] (Fig. 3A).

CRISPR interference screens were conducted on human-induced pluripotent stem cells (hiPSC)-derived astrocytes cultured with microglia-derived pro neuroinflammatory cytokines (TNF, IL-1α, and C1q) to determine relevant pathways propagating inflammatory astrocyte reactivity [32]. IL-6 and interferon signaling downstream of canonical NF-κB activation drove two distinct inflammatory reactive signatures, and both are modulated by the signal transducer and activator of transcription 3 (STAT3). These signatures were validated in both mouse models and in human AD brains [32]. In addition, genes involved in the mammalian target of rapamycin (mTOR) pathway were found to be significantly changed [32]. In a follow-up study by the same group, CRISPR interference screens were conducted on human iPSC-derived astrocytes treated with pro-inflammatory cytokines (e.g., TNF, IL-1α, and C1q) to identify molecular targets that affects lysosomal acidification dysfunction and exocytosis [33]. First, significant lysosomal alkalization and autophagic dysfunction were observed in neurotoxic reactive astrocytes. Lysosomal alkalization has been attributed to multiple downregulated genes including V-ATPase subunits and lysosomal hydrolases [33]. Importantly, it was found that mTOR is a central upstream regulator of this phenotype which is linked to lysosomal acidification dysfunction in inflammatory reactive astrocytes [33]. Inhibition of mTOR restored lysosomal acidification and rescued this phenotype associated with neurodegenerative diseases [33]. Moreover, these reactive astrocytes have increased lysosome exocytosis, leading to the secretion of toxic materials. In inflammatory reactive astrocytes, mTOR activation remodels lysosomal functions and induces unconventional secretion of IL-32 which is involved in the polarization of astrocyte reactive states [35], while mTOR inhibition reduces the intracellular levels and secretion of lipocalin-2 [36]. However, the relationship between whether the secreted materials directly mediate neuronal toxicity or if the materials contribute to toxicity through an indirect mechanism such as autocrine-paracrine signaling, remains to be elucidated. Emerging evidence indicates that mTOR activity is age-dependent, with aged and senescent astrocytes exhibiting distinct transcriptional profiles compared to other astrocytic populations [37, 38]. In aging astrocytes, reduced mTOR activity disrupts autophagy and lysosomal function, leading to protein trafficking defects and impaired synapse regulation. A subset of aging astrocytes, termed autophagy-dysregulated astrocytes, displays lysosomal dysfunction, abnormal autophagosome accumulation, and impaired proteasome function, resulting in synapse loss and reduced dendritic spines [37]. While senescent astrocytes exhibit proinflammatory profiles partly driven by active mTOR signaling and DNA-damage response pathways [38], these findings highlight the shifting roles of mTOR in aging astrocytes and its potential as a therapeutic target in neurodegenerative diseases.

In other disease contexts, it was shown that the stimulation of toll-like receptor 3 (TLR3) triggers lysosomal alkalization and release of adenosine triphosphate (ATP) and luminal contents from optic nerve head astrocytes [39]. In MS patients and mice, a subset of astrocytes that expresses the lysosomal protein LAMP1 and the TNF-related apoptosis-inducing ligand (TRAIL) has been identified [40], bridging the link between lysosomal dysfunction and astrocyte-mediated neuroinflammation. In a lysosomal storage disease model of astrocytes, there is progressive neuroinflammatory response and inhibition of autophagic function [41]. In addition, in Gaucher disease patients derived induced astrocytes, there is reduced glucocerebrosidase activity, cathepsin D activity, and increased inflammatory response [42]. In cortical astrocytes isolated from mice with multiple sulfatase deficiency, there is impaired lysosomal/autophagic dysfunction and accumulation of autophagic substrates [43]. There are also examples of activation of autophagy functions in astrocytes to attenuate inflammasome activation or inflammatory phenotypes in neurodegenerative diseases [44, 45]. Hence, the crosstalk between autolysosomal acidification dysfunction and neuroinflammation, and their pathogenic roles in neurodegenerative and neuroimmune disorders warrant further investigations. It is important to design future studies around the goal of examining the association between autolysosomal acidification dysfunction and neuroinflammation in heterogenous cell cultures or in vivo.

## Metabolic stressors

Astrocytes and neurons operate as a tightly coupled unit for energy metabolism in the brain. As neurons expend a considerable amount of ATP on neurotransmission, astrocytic mitochondrial metabolism can release signaling molecules like ATP and glutamate, allowing neurons to allocate more cellular resources to sustain high activity rates during information processing [46, 47]. Dysfunctional astrocytic mitochondria can lead to impaired glutamate clearance and increased levels of extracellular glutamate, ultimately inducing glutamate toxicity in neighboring neurons [48]. Furthermore, impaired mitochondria in astrocytes can also lead to increased ROS production or mitochondrial-derived damage-associated molecular patterns into the cytoplasm, which can also result in neuronal death [48, 49]. Hence, maintaining astrocytic mitochondrial function is essential to protect against neurodegeneration. Importantly, optimal lysosomal acidification maintains the turnover of damaged mitochondria through autophagic clearance, closely regulating mitochondrial metabolism in astrocytes [50, 51]. On the other hand, mitochondria regulate lysosomal pH by supplying ATP to activate the V-ATPase to acidify the lysosomal lumen [52]. Therefore, understanding and maintaining proper mitochondria-lysosome crosstalk is crucial to regulating astrocytic function (Fig. 3B).

Chronic exposure to diet derived metabolites like homocysteine and glucose affect astrocyte function. Exposure to homocysteine, a homologue of the amino acid cysteine, in astrocytes leads to lysosomal and autophagic impairments, including lysosomal acidification dysfunction due to downregulation of V-ATPase [53]. This further leads to increased oxidative stress and astrocytic cell death [53]. Chronic exposure to high levels of glucose in astrocytes induce mitochondrial oxidative stress through 5’AMP-activated protein kinase-independent pathways and lead to inhibition of transcription factor EB (TFEB) [54]. This consequently leads to impaired lysosomal acidification, reduced phagocytic function, and accumulation of oligomeric Aβ in astrocytes [54]. Exposure to high glucose has also been reported to promote ferroptosis of astrocytes by disrupting iron metabolism, which can be rescued by administration of gemfibrozil, a peroxisome proliferator-activated receptor alpha (PPARα) agonist. Gemfibrozil prevented the accumulation of lipid peroxidation products and ROS induced by iron deposition in astrocytes and inhibited ferroptosis of astrocytes [55]. Gemfibrozil has been shown to upregulate TFEB and enhance lysosomal biogenesis in astrocytes via PPARα [55], suggesting that lysosomal acidification may play an important role in alleviating high glucose induced metabolic dysfunction in astrocytes.

In a brain ischemia mouse model, increased expression of inflammatory cytokines TNF and IL-1β was seen in astrocytes. Using an oxygen-glucose deprivation/reoxygenation (OGD/R) model of primary mouse astrocytes to study brain ischemia in vitro, lysosomal impairment and autophagic dysfunction were observed, as determined by decreased lysosome number, increased lysosomal size, and accumulation of autophagosome associated proteins [56]. In a OGD primary rat astrocytes model, it was shown that there is an increase in lysosomal membrane permeabilization (LMP) and cathepsin release from lysosomes into the cytoplasm of astrocytes, leading to cell death [57]. This was also seen in another study using a similar model [58], alongside with disrupted mitochondrial membrane potential, increased production of ROS and inflammatory cytokines TNF, IL-6 and FasL, as well as apoptosis in astrocytes [58]. The knockdown of receptor interacting protein 1, an essential molecule in mediating TNF signaling, blocked OGD-induced increase in LMP and astrocyte death, suggesting that TNF downstream pathways contribute to lysosomal dysfunction in ischemic astrocytes [57]. In another instance, conditional knockdown of LAMP-2 A in ischemic astrocytes inhibited their activation and prevented the translocation of the pro-apoptotic proteins Bax and Bad to mitochondria, thereby preventing neuronal death, suggesting that elevated astrocytic LAMP-2 A contributes to ischemic vulnerability.

## Myelin and lipid induced toxicity and dysfunction

Astrocytes are actively involved in lipid metabolism in the brain and can be affected by increased levels of saturated fatty acids associated with dysfunctional lipid metabolism, neuronal myelin damage, and obesity [59–61]. Cultured brain astrocytes present higher capacity to process lipids in their oxidative metabolism and have a higher propensity to uptake fatty acids than other cell types in the brain [62]. The sensitivity of astrocytes and their reactive state transformation under lipid-induced oxidative stress such as sphingosine-1-phosphate (S1P) and apolipoprotein E (*APOE*) mutations, myelin debris accumulation and chronic exposure to fatty acids could lead to failure in their functions (Fig. 3C). S1P is a bioactive signaling lipid involved in several vital processes, including cellular proliferation, survival, and migration. Autosomal recessive mutations in sphingosine-1-phosphate lyase 1 which encodes for S1P lyase, leads to neurodevelopmental disorders. An excess of S1P due to mutations in S1P lyase led to increased activity of regulatory enzymes involved in the tricarboxylic acid cycle and increased cellular ATP content, which subsequently activated mTOR and reduced lysosomal-autophagosome fusion as well as reduced autophagic function of astrocytes [63]. Phospholysine phosphohistidine inorganic pyrophosphate phosphatase (LHPP) is another enzyme that is highly expressed in the brain which catalyzes pyrophosphate to orthophosphate. LHPP is primarily expressed in the lysosomes of astrocytes and has optimal enzymatic activity at an acidic pH [64]. Under stress conditions, LHPP modulates lysosomal acidification through pyrophosphate hydrolysis driven proton transport through the V-ATPase, thereby averting the adverse impact of chronic stress on adult hippocampal neurogenesis [64].

Apolipoprotein E (ApoE) plays a major role in cholesterol and phospholipid regulation within the CNS [65] and astrocytes are the primary source of ApoE in the brain [66]. The E4 allele of *APOE* (*APOE4*) is the strongest genetic risk factor for the development of late onset AD. *APOE4* astrocytes accumulate high amounts of lipid droplets and have decreased fatty acid uptake and oxidation compared to *APOE3* astrocytes [67]. This observation is also consistent with other reports related to lysosomal dysfunctions associated with *APOE4* astrocytes [68], with a specific study illustrating that lysosomes in *APOE4* astrocytes have a higher lysosomal pH than *APOE3* astrocytes [69]. *APOE*4 also downregulates the sodium-hydrogen exchanger 6, which create dysregulation of the endosomal pH in astrocytes, leading to Aβ accumulation within the astrocytes [70]. The impairments of endolysosomal and autophagic function in *APOE4* astrocytes has also been reported in other studies [69, 71, 72]. In addition, *APOE4* astrocytes induce cholesterol accumulation that impairs lysosomal turnover of damaged mitochondria and treatment of therapeutic agents that remove cholesterol restores autophagic and mitochondrial activity [73].

In the CNS, myelin uptake is thought to occur primarily by microglia. However, recent studies have shown that astrocytes also participate in this process [74, 75]. Astrocytes actively phagocytose myelin debris during demyelination [76, 77]. In a study using primary rat astrocytes culture, the myelin debris was taken up by astrocytes through receptor-mediated endocytosis and are transported to lysosomes for degradation. Exposure to excessive myelin debris resulted in astroglial nuclear factor kappa B (NF-κB) activation and secretion of inflammatory cytokines [76]. These findings were confirmed in the context of MS, where myelin-positive astrocytes had increased nuclear localization of NF-κB and cytokine expression compared to astrocytes lacking myelin [76]. In a similar astrocyte culture model of spinal cord injury, engulfed myelin debris are transported to lysosomes for degradation and led to an increase in lysosomal size, which consequently resulted in excessive glia scar formation [77]. Apart from myelin debris accumulation, accumulation of cholesterol can also lead to impairment of lysosomal acidification and increased lysosomal leakiness in foamy phagocytes, which can eventually lead to cell death [78].

Chronic exposure of fatty acid (e.g., palmitic acid) in astrocytes have led to intracellular lipid accumulation with an elevation of the pH in the lysosomes along with autophagic dysfunction, and an increase in the mRNA expression of pro-inflammatory cytokines [79]. High-fat diet (HFD) feeding induces peripheral obesity which develops inflammation and has been shown to affect CNS function [80, 81]. In mice under HFD, there is increased hypothalamic inflammatory signaling, reactive astrogliosis and microgliosis, along with neuronal injury [82]. In a similar study, HFD feeding to mice reduced mitochondrial number and increased mitochondrial size, thereby contributing to reduced activity of hypothalamic astrocytes [83]. In addition, there are other models of liver related injury or inflammation that has been found to lead to neurodegeneration [80]. For instance, in alcoholic liver disease mouse model, exposure to ethanol impaired lysosomal acidification and function in astrocytes and reduced autophagic function [84]. In a hepatic encephalopathy model where mice are exposed to high levels of ammonia, astrocytes had decreased lysosomal acidification and increased accumulation of ROS [85], leading to neuronal toxicity. ROS has been shown to further interact with lysosomal membranes through peroxidation, which destabilizes the membrane [86], leading to decreased efficacy of proton pumps and pH “leaking”, which would impair lysosomal acidification [86, 87].

## Toxic protein aggregates

Astrocytes play a key role in the clearance of toxic protein aggregates that are hallmarks of many neurodegenerative diseases [88–91]. However, they are still vulnerable to the toxic effects of protein aggregation in both familial and sporadic pathologies, which have been implicated in lysosomal dysfunction, autophagic inhibition, and propagation of neurodegenerative pathology (Fig. 3D). In AD, accumulation of toxic aggregates such as amyloid beta (Aβ) has been attributed to astrocytes dysfunction [92]. Presenilin-1 (PS1) mutation has been shown to impair lysosomal vesicle trafficking, leading to reduced degradation capacity, and accumulation of Aβ fibrils in astrocytes [93]. *APP/PS1* transgenic mice displayed a higher density of astrocytes and increased accumulation of lysosomes in cells, potentially due to a higher phagocytic activity required to clear a higher burden of toxic materials in the mice [94]. The stimulation of lysosomal biogenesis with TFEB in astrocytes has been shown to reduce Aβ plaque load in the hippocampus of *APP/PS1* mice [95]. Another study has shown that a small molecule agonist of angiotensin-(1–7) receptor (AVE 0991) could suppress astrocyte neuroinflammatory responses by enhancing autophagy. Treatment of AVE 0991 reduced Aβ deposition as well as rescued neuronal death and cognitive deficits in *APP/PS1* mice [96].

Human iPSC-derived astrocytes or primary astrocytes exposed to Aβ fibrils or oligomers showed accumulation of Aβ inclusions that were enclosed within LAMP1-positive lysosomes and sustained markers associated with reactivity [97, 98]. Additionally, Aβ uptake and accumulation in astrocytes resulted in endoplasmic reticulum and mitochondrial swelling, autolysosomal dysregulation, LMP, formation of pathological lipid structures, and increased secretion of chemokines and cytokines [97, 99, 100]. Furthermore, increasing evidence have pointed to the role of astrocytes in Aβ fibrils uptake and aggregation, leading to indirect neurotoxic qualities [101, 102]. Specifically, high level of glycoprotein YKL-40 in astrocytes can promote neurotoxicity and YKL-40 knockout astrocytes exhibit enhanced lysosomal acidification as well as increased uptake and degradation of Aβ peptides [103]. In a study using neuron-astrocyte co-cultures, it was shown that incomplete astrocyte phagocytosis of Aβ fibrils leads to increased astrocytic secretion of toxic vesicles as well as accumulation of Aβ fibrils in neurons and neuronal cell death [101]. Ineffective clearance in astrocytes is possibly attributed to increased levels of Rab27a protein, which reduces lysosomal acidity through Nox2 recruitment [101]. In addition, increasing levels of autophagic flux in astrocytes via progesterone has been shown to be effective in enhancing the neuroprotective and anti-inflammatory effect of astrocytes in models of AD [104]. Furthermore, stimulation of autophagy with Sirtuin-1 in primary rat astrocytes has also been shown to improve lysosomal function through upregulation of V-ATPase subunits and increase in lysosome number, leading to more effective clearance of Aβ fibrils [105].

In primary human astrocytes exposed to preformed 4R tau fibrils, the endocytosis of tau aggregates causes lysosomal swelling, permeabilization, and lysosomal deacidification [106]. In primary mouse astrocytes treated with tau pre-formed fibrils, expression of TFEB enhanced lysosomal activity, increased tau degradation and inhibited tau transmission [107]. TFEB activation enhances the phagocytic capacity of astrocytes, through increasing the uptake of pre-formed fibrils, and increases the incidence of phagocytosed pre-formed fibrils inside the lysosome, indicating that TFEB enhances both uptake and degradation of phagocytosed proteins [107]. In addition, astroglial TFEB overexpression reduced tau pathology, spreading, and gliosis in the hippocampus of PS19 tauopathy mice [107]. Studies in another tauopathy model using rTg4510 tau transgenic mouse have also shown that TFEB expression enhanced lysosomal activity and clearance of autophagic substrates and phosphorylated tau [108]. Another study from the same group highlights TFEB’s role in mediating the lysosomal exocytosis of mutant truncated tau, both in vitro and in PS19 transgenic mice. This process, dependent on the lysosomal target TRPML1, is positively correlated with tau clearance. Loss of TFEB increases tau pathology and spreading, suggesting that TFEB-mediated lysosomal exocytosis of tau acts as a clearance mechanism to reduce intracellular tau under pathological conditions [109].

In PD, α-synuclein is the major component of neuronal cytoplasmic aggregates called Lewy bodies, which are the main pathological hallmark of the disease. In immortalized astrocyte cell lines, overexpression of wild-type α-synuclein as well as A30P and A53T mutant α-synuclein led to inhibition of autophagy, loss of mitochondrial membrane potential, and cell death [110]. Primary astrocytes with A53T α-synuclein overexpression or treatment with α-synuclein aggregates had decreased lysosomal acidification and reduced lysosomal enzyme activity, thereby contributing to the release of more extracellular vesicles which propagate PD pathology [111]. Incubation of astrocytes with Lewy body extracts from human PD patients or α-synuclein preformed fibrils led to α-synuclein colocalization in lysosomes, indicating aggregate buildup due to reduced lysosomal degradative capacity [112, 113]. Additionally, there is increased mitochondrial driven cytotoxicity in astrocytes [112]. Dose dependent treatment with lysosomal V-ATPase inhibitor Bafilomycin A1 led to an increase in the accumulation of α-synuclein fibrils in astrocytes, indicating that lysosomal acidification plays an important role in modulating α-synuclein buildup in astrocytes [114]. Increasing levels of autophagic flux in astrocytes via rapamycin have been shown to be effective in enhancing the neuroprotective and anti-inflammatory effect of astrocytes in models of PD [115]. In other types of familial PD, mutations in *LRRK2*, *ATP13A2*, *GBA1*, and *PARK7* impair lysosomal function and degradative capacity of astrocytes [42, 116–118]. *LRRK2* G2019S primary mouse astrocytes have enlarged lysosomes and abnormal lysosomal pH, which led to reduced lysosomal activity, and is regulated by LRRK2 localization to lysosomes [116]. Inhibition of LRRK2 kinase activity with PF-06447475 restored defects in lysosomal morphology and function [116]. *ATP13A2* mutations in astrocytes resulted in decreased lysosomal proteolysis function and increased accumulation and propagation of α-synuclein [117]. Patient derived induced astrocytes with *GBA* mutations also exhibited impaired lysosomal enzyme activity, leading to α-synuclein accumulation [42]. In DJ1 knockout iPSC-derived midbrain organoid models, impaired lysosomal proteolysis results in increased α-synuclein phosphorylation, protein aggregation, and the accumulation of advanced glycation end products. Astrocytes play a role in these effects, as DJ1 loss diminishes their metabolic support capacity and promotes a pro-inflammatory phenotype. In co-culture models, DJ1-expressing astrocytes have been shown to rescue proteolysis deficits [118].

## Restoration of lysosomal acidification as a therapeutic target

Functional lysosomal V-ATPase and ion channels such as two-pore channels (TPC) are crucial in maintaining lysosomal acidification and function of astrocytes [119]. In rat astrocytes, activation of TPC by nicotinic acid adenine dinucleotide phosphate (NAADP) increases autophagosome and lysosome formation [119]. To enhance the function of lysosomal V-ATPase, C381 is a small-molecule activator of V-ATPase that has been applied to promote lysosomal acidification in microglia [120], although its effect in astrocytes remains to be tested. OSI-027 and PP242 are two other small-molecular mTOR inhibitors that have been identified by high-throughput screening using a fluorescent protein based lysosomal pH biosensor [121]. OSI-027 and PP242 were identified as the top lysosome-acidifying hits in human iPSC-derived astrocytes which demonstrated increased lysosomal cathepsin activity and improved autophagic function [121]. In primary astrocytes under exposure to environmental toxins, lysosomal acidification and autophagic flux are impaired and can be restored by PP242 treatment [122]. Recent developments using lysosomal-acidifying nanoparticles to target and restore acidification of impaired lysosomes have been demonstrated in astrocytes [23, 24]. The introduction of lysosomal-acidifying nanoparticles to mouse primary astrocytes has led to increased lysosomal acidification which increased the lysosomal cathepsin activity and astrocytic phagocytosis of cell debris [23, 24]. Other types of lysosomal-acidifying nanoparticles have also been developed [123–125] to re-acidify impaired lysosomes and promote autophagic degradation, and this has been reviewed elsewhere [126–128].

Another approach to promote lysosomal acidification is through increasing cyclic adenosine monophosphate (cAMP) levels [129]. Bafilomycin A1 treatment to astrocytes induced dysfunctional V-ATPase and lysosomal alkalization, while increasing cAMP levels via activation of PKA signaling pathway restored lysosomal acidification [130]. Treatment with cilostazol, a phosphodiesterase inhibitor that inhibits the degradation of cAMP, reacidifies lysosomes in astrocytes, thereby increasing Aβ degradation in astrocytes [131]. In addition, while the acute treatment of cAMP activates the AKT survival pathway in astrocytes, chronic exposure of cAMP has been observed to activate the FoxO-mediated Bim/Bax death pathway [132]. Therefore, the reliance on cAMP elevation to acidify lysosomes requires dosage optimization, as high cAMP level is observed to exacerbate the vulnerability of astrocytes to oxidative stress [132, 133]. Interestingly, pharmacological inhibitors and siRNAs of H^+^/K^+^-ATPase elevated lysosomal pH in bafilomycin A1 and cAMP co-treated astrocytes, suggesting that H^+^/K^+^-ATPase may function as an alternative proton pump for lysosomes when the V-ATPase function is impaired [130]. Hence, pharmacological agents that target the H^+^/K^+^-ATPase may be a new avenue for lysosome-acidifying therapeutics. Other molecular targets to restore lysosomal pH and autophagic function have been explored. An important therapeutic target is TFEB, where its expression regulates lysosome biogenesis and expression of V-ATPase, thereby maintaining lysosomal acidification [107, 134]. Stereotaxic injection of adeno-associated viral particles carrying *TFEB* driven by a glial fibrillary acidic protein (GFAP) promoter was used to achieve astrocyte-specific expression of the gene in the hippocampus of *APP/PS1* transgenic mice. Expression of TFEB in these astrocytes enhanced lysosome function, resulting in reduced Aβ plaques in the hippocampus [95]. Aspirin has also been shown to upregulate TFEB and increases lysosomal biogenesis in mouse astrocytes through inducing the activation of PPARα and stimulated the transcription of TFEB [135]. Furthermore, progranulin may also be a promising target as it mediates TFEB expression [95, 136]. However, careful regulation of TFEB expression is essential, as excessive activation could lead to potential side effects, including its role as an oncogenic regulatory marker [137, 138].

## Summary and future perspectives

**Fig. 4:**
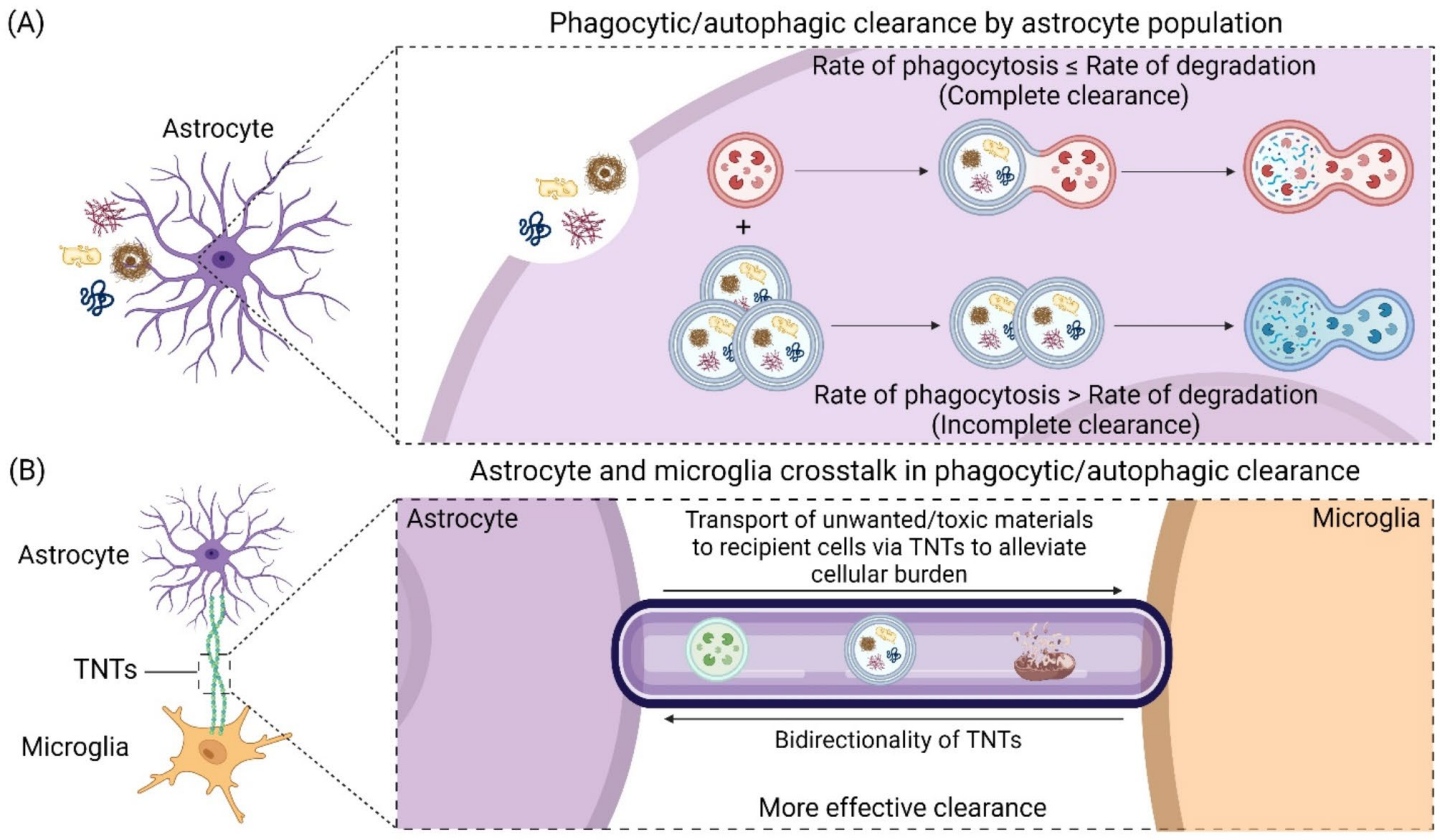
Astrocyte-microglia interactions to promote clearance of unwanted and toxic materials in the cells. (**A**) Under normal conditions where the unwanted materials are within the clearance capacity of astrocytes, phagocytic/autophagic degradation of the accumulated materials can proceed. During chronic exposure to these unwanted materials, lysosomal functions are impaired, and this reduces the capacity of astrocytes to degrade, leading to the accumulation of toxic materials within the astrocytes and their subsequent dysfunction. (**B**) Astrocytes-microglia crosstalk through tunneling nanotubes (TNTs). In astrocyte-microglia interaction, unwanted and toxic cellular materials such as impaired lysosomes, undegraded autophagosomes, and damaged mitochondria can migrate to microglia, where the latter can assist in more effective degradation of these toxic products. The figure was created with BioRender.com

Astrocytes play a critical role in maintaining energy metabolism and neuronal health in the CNS [4, 139]. The effectiveness of autophagic and phagocytic functions by astrocytes depends on the extent of lysosomal acidification and degradation (Fig. 4A). Pathogenic factors, including neuroinflammatory signaling, metabolic stressors, and the accumulation of lipids and toxic protein aggregates, contribute to the impairment of astrocytic lysosomal acidification, resulting in dysfunctional autophagy and phagocytosis. However, it remains unclear whether these pathogenic factors directly drive lysosomal dysfunction in astrocytes or if the defects are secondary effects of astrocyte reactivity induced by these stressors, neither is mutually exclusive. Further studies are required to disentangle these mechanisms and clarify the causal relationships underlying astrocytic dysfunction. Due to the crosstalk between mitochondria and lysosome [140], it is important to investigate how restoration of mitochondria function, metabolic activity, and lipid metabolism in astrocytes might offer potential avenues to maintain optimal lysosomal acidification and effective degradation.

The generation of the mRFP-eGFP-LC3 mouse model, which is designed to monitor changes in autophagic flux due to alterations in lysosomal acidification [141], with a astrocyte promoter such as GFAP will enable the examination of the effects of lysosomal acidification impairment in astrocytes in vivo. We also discussed molecular targets and potential therapeutics that regulate lysosomal pH, including small molecules and lysosome-targeting nanoparticles. It is essential to further investigate how these therapeutics modulate lysosomal acidification and overall cellular function under different disease conditions and reactivity states of astrocytes. Furthermore, profiling of astrocyte heterogeneity using omics characterizations will reveal cellular subtypes that are directly implicated by lysosomal acidification dysfunction and enable the elucidation of new molecular targets for better and more effective intervention [32, 142–145]. While this review focuses on targeting lysosomal acidification in astrocytes, it is important to note that lysosomal dysfunction also occurs in other CNS cell types, such as microglia and neurons, under pathological conditions [127, 128, 146]. In addition to defects in lysosomal acidification, lysosomal enzyme deficiencies play a critical role in maintaining astrocytic function, as well as the function of other CNS cell types [147–149]. In infantile neuronal ceroid lipofuscinosis (CLN1 disease), primary cultures of astrocytes, microglia, and neurons derived from Ppt1-deficient mice exhibit impaired cellular function. Ppt1-deficient astrocytes display dysregulated calcium signaling, resulting in increased cell death. In co-culture experiments, the presence of both Ppt1-deficient astrocytes and microglia further disrupted the morphology of both wild-type and Ppt1-deficient neurons [150], suggesting that the astrocytes be cross primed by impaired microglia to become neurotoxic under disease conditions [150, 151]. Given the interconnected roles of these cells, a broader therapeutic approach targeting lysosomal function across multiple CNS cell types may offer more comprehensive benefits, highlighting the need to assess the extent and specific cellular contributions of lysosomal impairments.

The supportive functions of astrocytes have recently been extended to them taking on a more active role as phagocytes similar to microglia. These functions include the phagocytosis of cellular debris, synapse elimination, and the regulation of neuronal activity [8, 152]. Comparative studies suggest that astrocytes complement the phagocytic activity of microglia, although their mechanisms of action are distinct [8, 152]. Astrocytic activity-dependent synaptic pruning requires the involvement of phagocytic receptors multiple EGF-like-domains 10 (MEGF10) and MER Tyrosine Kinase (MERTK), both of which are highly expressed in developing astrocytes [153]. Subsequent studies have highlighted the critical role of MEGF10 in synaptic pruning within adult mice, where astrocytes eliminate excitatory synapses in the hippocampus to maintain circuit homeostasis and support memory formation [154]. In contrast, microglia-mediated synapse elimination involves activation of the classical complement pathway [155–157]. Specifically, the complement cascade initiator, C1q, localizes to developing synapses, marking them for microglial phagocytosis in a complement component 3-dependent manner [155]. A recent study illustrates that the lysosomal pH of astrocytes is lower than that of microglia and astrocytes are more resistant to alterations in lysosomal pH compared to microglia [158]. Despite being more acidic, another study has shown that astrocytes phagocytose less Aβ than microglia in cell culture and rat brain slices [159]. In the presence of dying cells, astrocytes appear to degrade most cells at proximity without the need for constant cell migration, while microglia are sparse and require constant movement to detect and engage dying cells. This suggests that astrocytic phagocytosis would be more energetically favorable than microglia [160]. A similar study in the degradation of toxic materials like Aβ show a complementary feedback between microglia and astrocytes to remove the aggregate [161]. Furthermore, there is evidence to suggest that astrocytes can potentially exchange materials with microglia through tunneling nanotubes (Fig. 4B), which may promote more efficient phagocytosis, although the exact mechanism remains to be investigated [162–164].

An important future direction of study would be to document crosstalk between microglia and astrocytes, which would provide more detailed understanding and insights into the overall phagocytic processes [165, 166]. This new appreciation for the phagocytic function of astrocytes complements the basal autophagy functions of astrocytes to contribute to neurodegenerative and neuroprotective mechanisms in the CNS. This shifts the current treatment paradigm to consider restoration of lysosomal acidification and degradative functions in astrocytes as a therapeutic target for neurodegenerative diseases [24, 95]. Astrocytes and microglia may have cooperative or opposing interactions during phagocytosis as well as further interactions with neurons [167, 168]. The extent of which they target specific synapses or toxic proteins and how they work together in different circumstances requires further investigation. There is also interaction between astrocytes and endothelial cells through microRNA that targets V-ATPase and modulates lysosomal acidification which can determine the level of endothelial adhesion molecules and the extent of neutrophil migration through the BBB [169]. It is important to comprehend these interactions and their effects on brain homeostasis under both healthy and diseased conditions, as this knowledge is crucial for developing treatments for neurological disorders.

## Data Availability

No datasets were generated or analysed during the current study.

## References

[1] von Bartheld CS, Bahney J, Herculano-Houzel S. The search for true numbers of neurons and glial cells in the human brain: A review of 150 years of cell counting. J Comp Neurol. 2016. pp. 3865–95. 10.1002/cne.24040 PMC5063692 27187682

[2] Valles SL, Singh SK, Campos-Campos J, Colmena C, Campo-Palacio I, Alvarez-Gamez K et al. Functions of astrocytes under normal conditions and after a brain disease. Int J Mol Sci. 2023. 10.3390/ijms24098434 PMC10179527 37176144

[3] Pociūtė A, Pivoriūnas A, Verkhratsky A. Astrocytes dynamically regulate the blood-brain barrier in the healthy brain. Neural Regeneration Res. 2024;19. 10.4103/1673-5374.382248 PMC10664108 37843196

[4] Chen Z, Yuan Z, Yang S, Zhu Y, Xue M, Zhang J, et al. Brain energy metabolism: astrocytes in neurodegenerative diseases. CNS Neurosci Ther. 2023;29:24–36. 36193573 10.1111/cns.13982 PMC9804080

[5] Park J, Chung W-S. Astrocyte-dependent circuit remodeling by synapse phagocytosis. Curr Opin Neurobiol. 2023;81:102732. 37247606 10.1016/j.conb.2023.102732

[6] Ponath G, Park C, Pitt D. The role of astrocytes in multiple sclerosis. Front Immunol. 2018;9:217. 29515568 10.3389/fimmu.2018.00217 PMC5826071

[7] Xu T, Liu C, Deng S, Gan L, Zhang Z, Yang G-Y, et al. The roles of microglia and astrocytes in Myelin phagocytosis in the central nervous system. J Cereb Blood Flow Metabolism. 2022;43:325–40. 10.1177/0271678X221137762 PMC9941857 36324281

[8] Konishi H, Koizumi S, Kiyama H. Phagocytic astrocytes: emerging from the shadows of microglia. Glia. 2022;70:1009–26. 35142399 10.1002/glia.24145 PMC9305589

[9] Giovannoni F, Quintana FJ. The role of astrocytes in CNS inflammation. Trends Immunol. 2020;41:805–19. 32800705 10.1016/j.it.2020.07.007 PMC8284746

[10] Hasel P, Aisenberg WH, Bennett FC, Liddelow SA. Molecular and metabolic heterogeneity of astrocytes and microglia. Cell Metabol. 2023;35:555–70. 10.1016/j.cmet.2023.03.006 36958329

[11] Lo CH, Skarica M, Mansoor M, Bhandarkar S, Toro S, Pitt D. Astrocyte heterogeneity in multiple sclerosis: current Understanding and technical challenges. Front Cell Neurosci. 2021;15:726479. 34456686 10.3389/fncel.2021.726479 PMC8385194

[12] Matias I, Morgado J, Gomes FCA. Astrocyte heterogeneity: impact to brain aging and disease. Front Aging Neurosci. 2019. 10.3389/fnagi.2019.00059 PMC6433753 30941031

[13] Moulson AJ, Squair JW, Franklin RJM, Tetzlaff W, Assinck P. Diversity of Reactive Astrogliosis in CNS Pathology: Heterogeneity or Plasticity? Frontiers in Cellular Neuroscience. 2021. 10.3389/fncel.2021.703810 PMC8349991 34381334

[14] Brandebura AN, Paumier A, Onur TS, Allen NJ. Astrocyte contribution to dysfunction, risk and progression in neurodegenerative disorders. Nat Rev Neurosci. 2023;24:23–39. 36316501 10.1038/s41583-022-00641-1 PMC10198620

[15] Kreher C, Favret J, Maulik M, Shin D. Lysosomal functions in glia associated with neurodegeneration. Biomolecules. 2021;11. 10.3390/biom11030400 PMC7999372 33803137

[16] Sung K, Jimenez-Sanchez M. Autophagy in astrocytes and its implications in neurodegeneration. J Mol Biol. 2020;432:2605–21. 31931011 10.1016/j.jmb.2019.12.041

[17] Zhou Z, Zhou J, Liao J, Chen Z, Zheng Y. The emerging role of astrocytic autophagy in central nervous system disorders. Neurochem Res. 2022. 10.1007/s11064-022-03714-w 35960484

[18] Galloway DA, Phillips AEMM, Owen DRJJ, Moore CS. Phagocytosis in the brain: homeostasis and disease. Front Immunol. 2019;10:790. 31040847 10.3389/fimmu.2019.00790 PMC6477030

[19] Wang J-L, Xu C-J. Astrocytes autophagy in aging and neurodegenerative disorders. Biomed pharmacotherapy = Biomedecine Pharmacotherapie. 2020;122:109691. 10.1016/j.biopha.2019.109691 31786465

[20] Kawai A, Uchiyama H, Takano S, Nakamura N, Ohkuma S. Autophagosome-lysosome fusion depends on the pH in acidic compartments in CHO cells. Autophagy. 2007;3:154–7. 17204842 10.4161/auto.3634

[21] Papouin T, Dunphy J, Tolman M, Foley JC, Haydon PG. Astrocytic control of synaptic function. Philosophical Trans Royal Soc Lond Ser B Biol Sci. 2017;372. 10.1098/rstb.2016.0154 PMC5247586 28093548

[22] Kıray H, Lindsay SL, Hosseinzadeh S, Barnett SC. The multifaceted role of astrocytes in regulating myelination. Exp Neurol. 2016;283:541–9. 26988764 10.1016/j.expneurol.2016.03.009 PMC5019113

[23] Lööv C, Mitchell CH, Simonsson M, Erlandsson A. Slow degradation in phagocytic astrocytes can be enhanced by lysosomal acidification. Glia. 2015;63:1997–2009. 26095880 10.1002/glia.22873 PMC6728804

[24] Lööv C, Erlandsson A. Lysosomal acidification in cultured astrocytes using nanoparticles. Methods in molecular biology. (Clifton NJ). 2017;1594:165–77. 10.1007/978-1-4939-6934-0_10 28456982

[25] Cox GM, Kithcart AP, Pitt D, Guan Z, Alexander J, Williams JL, et al. Macrophage migration inhibitory factor potentiates Autoimmune-Mediated neuroinflammation. J Immunol. 2013;191:1043–54. 23797673 10.4049/jimmunol.1200485

[26] Quan L, Uyeda A, Muramatsu R. Central nervous system regeneration: the roles of glial cells in the potential molecular mechanism underlying remyelination. Inflamm Regeneration. 2022;42:7. 10.1186/s41232-022-00193-y PMC8888026 35232486

[27] Kallergi E, Siva Sankar D, Matera A, Kolaxi A, Paolicelli RC, Dengjel J, et al. Profiling of purified autophagic vesicle degradome in the maturing and aging brain. Neuron. 2023;111:2329–e23477. 37279748 10.1016/j.neuron.2023.05.011

[28] Litwiniuk A, Juszczak GR, Stankiewicz AM, Urbańska K. The role of glial autophagy in Alzheimer’s disease. Mol Psychiatry. 2023;28:4528–39. 37679471 10.1038/s41380-023-02242-5

[29] Mitsui S, Yamaguchi J, Suzuki C, Uchiyama Y, Tanida I. TUNEL-positive structures in activated microglia and SQSTM1/p62-positive structures in activated astrocytes in the neurodegenerative brain of a CLN10 mouse model. Glia. 2023;71:2753–69. 37571859 10.1002/glia.24449

[30] Wang T, Sun Y, Dettmer U. Astrocytes in Parkinson’s Disease: From Role to Possible Intervention. Cells. 2023. 10.3390/cells12192336 PMC10572093 37830550

[31] Liddelow SA, Guttenplan KA, Clarke LE, Bennett FC, Bohlen CJ, Schirmer L, et al. Neurotoxic reactive astrocytes are induced by activated microglia. Nature. 2017;541:481–7. 28099414 10.1038/nature21029 PMC5404890

[32] Leng K, Rose IVL, Kim H, Xia W, Romero-Fernandez W, Rooney B, et al. CRISPRi screens in human iPSC-derived astrocytes elucidate regulators of distinct inflammatory reactive States. Nat Neurosci. 2022;25:1528–42. 36303069 10.1038/s41593-022-01180-9 PMC9633461

[33] Rooney B, Leng K, Mccarthy F, Rose IVL, Herrington KA, Bax S et al. mTOR controls neurotoxic lysosome exocytosis in inflammatory reactive astrocytes. 2021;1–32.

[34] Lo CH, Zeng J, Loi GWZ, Saipuljumri EN, O’Connor LM, Indajang J et al. Defective lysosomal acidification contributes to TNFR1 mediated neuronal necroptosis in Alzheimer’s disease. bioRxiv. 2023;2023.10.12.562041.

[35] Leng K, Rooney B, McCarthy F, Xia W, Rose IVL, Bax S, et al. mTOR activation induces endolysosomal remodeling and nonclassical secretion of IL-32 via exosomes in inflammatory reactive astrocytes. J Neuroinflamm. 2024;21:198. 10.1186/s12974-024-03165-w PMC11312292 39118084

[36] Jung B-K, Park Y, Yoon B, Bae J-S, Han S-W, Heo J-E, et al. Reduced secretion of LCN2 (lipocalin 2) from reactive astrocytes through autophagic and proteasomal regulation alleviates inflammatory stress and neuronal damage. Autophagy. 2023;19:2296–317. 36781380 10.1080/15548627.2023.2180202 PMC10351455

[37] Lee E, Jung Y-J, Park YR, Lim S, Choi Y-J, Lee SY, et al. A distinct astrocyte subtype in the aging mouse brain characterized by impaired protein homeostasis. Nat Aging. 2022;2:726–41. 37118130 10.1038/s43587-022-00257-1

[38] Simmnacher K, Krach F, Schneider Y, Alecu JE, Mautner L, Klein P, et al. Unique signatures of stress-induced senescent human astrocytes. Exp Neurol. 2020;334:113466. 32949572 10.1016/j.expneurol.2020.113466

[39] Beckel JM, Gómez NM, Lu W, Campagno KE, Nabet B, Albalawi F et al. Stimulation of TLR3 triggers release of lysosomal ATP in astrocytes and epithelial cells that requires TRPML1 channels. Sci Rep. 2018;8. 10.1038/s41598-018-23877-3 PMC5893592 29636491

[40] Sanmarco LM, Wheeler MA, Gutiérrez-Vázquez C, Polonio CM, Linnerbauer M, Pinho-Ribeiro FA, et al. Gut-licensed IFNγ + NK cells drive LAMP1 + TRAIL + anti-inflammatory astrocytes. Nature. 2021;590:473–9. 33408417 10.1038/s41586-020-03116-4 PMC8039910

[41] Di Malta C, Fryer JD, Settembre C, Ballabio A. Astrocyte dysfunction triggers neurodegeneration in a lysosomal storage disorder. Proc Natl Acad Sci USA. 2012;109:E2334–42. 22826245 10.1073/pnas.1209577109 PMC3435187

[42] Aflaki E, Stubblefield BK, McGlinchey RP, McMahon B, Ory DS, Sidransky E. A characterization of gaucher iPS-derived astrocytes: potential implications for Parkinson’s disease. Neurobiol Dis. 2020;134:104647. 31669751 10.1016/j.nbd.2019.104647 PMC6980699

[43] Di Malta C, Fryer JD, Settembre C, Ballabio A. Autophagy in astrocytes: a novel culprit in lysosomal storage disorders. Autophagy. 2012;8:1871–2. 23047468 10.4161/auto.22184 PMC3541309

[44] Dong A, Yang Y, Jiang S, Yao X, Qi D, Mao C, et al. Pramipexole inhibits astrocytic NLRP3 inflammasome activation via Drd3-dependent autophagy in a mouse model of Parkinson’s disease. Acta Pharmacol Sin. 2023;44:32–43. 35896696 10.1038/s41401-022-00951-1 PMC9813225

[45] Cheng X, Wei Y, Qian Z, Han L. Autophagy balances neuroinflammation in Alzheimer’s disease. Cell Mol Neurobiol. 2023;43:1537–49. 35960407 10.1007/s10571-022-01269-6 PMC11412430

[46] Ioannou MS, Jackson J, Sheu S-H, Chang C-L, Weigel AV, Liu H, et al. Neuron-Astrocyte metabolic coupling protects against Activity-Induced fatty acid toxicity. Cell. 2019;177:1522–e153514. 31130380 10.1016/j.cell.2019.04.001

[47] Volterra A, Meldolesi J. Astrocytes, from brain glue to communication elements: the revolution continues. Nat Rev Neurosci. 2005;6:626–40. 16025096 10.1038/nrn1722

[48] Bantle CM, Hirst WD, Weihofen A, Shlevkov E. Mitochondrial dysfunction in astrocytes: A role in Parkinson’s disease?? Front Cell Dev Biology. 2021;8. 10.3389/fcell.2020.608026 PMC7849831 33537300

[49] Lin M, Liu N, Qin Z, Wang Y. Mitochondrial-derived damage-associated molecular patterns amplify neuroinflammation in neurodegenerative diseases. Acta Pharmacol Sin. 2022;43:2439–47. 35233090 10.1038/s41401-022-00879-6 PMC9525705

[50] Deus CM, Yambire KF, Oliveira PJ, Raimundo N. Mitochondria-Lysosome crosstalk: from physiology to neurodegeneration. Trends Mol Med. 2020;26:71–88. 31791731 10.1016/j.molmed.2019.10.009

[51] Yazdankhah M, Ghosh S, Liu H, Hose S, Zigler JS, Sinha D. Mitophagy in Astrocytes Is Required for the Health of Optic Nerve. Cells. 2023. 10.3390/cells12202496 PMC10605486 37887340

[52] Demers-Lamarche J, Guillebaud G, Tlili M, Todkar K, Bélanger N, Grondin M, et al. Loss of mitochondrial function impairs lysosomes. J Biol Chem. 2016;291:10263–76. 26987902 10.1074/jbc.M115.695825 PMC4858975

[53] Tripathi M, Zhang CW, Singh BK, Sinha RA, Moe KT, DeSilva DA, et al. Hyperhomocysteinemia causes ER stress and impaired autophagy that is reversed by vitamin B supplementation. Cell Death Dis. 2016;7:e2513–2513. 27929536 10.1038/cddis.2016.374 PMC5260994

[54] Huang YC, Hsu SM, Shie FS, Shiao YJ, Chao L-JJ, Chen HW et al. Reduced mitochondria membrane potential and lysosomal acidification are associated with decreased oligomeric Aβ degradation induced by hyperglycemia: A study of mixed glia cultures. PLoS ONE. 2022;17. 10.1371/journal.pone.0260966 PMC8786178 35073330

[55] Wang N, Zhao Y, Wu M, Li N, Yan C, Guo H et al. Gemfibrozil alleviates cognitive impairment by inhibiting ferroptosis of astrocytes via restoring the Iron metabolism and promoting antioxidant capacity in type 2 diabetes. Mol Neurobiol. 2023. 10.1007/s12035-023-03589-0 37697219

[56] Luo D, Ye W, Chen L, Yuan X, Zhang Y, Chen C et al. PPARa Inhibits Astrocyte Inflammation Activation by Restoring Autophagic Flux after Transient Brain Ischemia. Biomedicines. 2023. 10.3390/biomedicines11030973 PMC10045980 36979952

[57] Du H, Guo Y, Zhu Y, Gao D, Lin B, Liu Y, et al. RIPK1 Inhibition contributes to lysosomal membrane stabilization in ischemic astrocytes via a lysosomal Hsp70.1B-dependent mechanism. Acta Pharmacol Sin. 2023;44:1549–63. 37055533 10.1038/s41401-023-01069-8 PMC10374908

[58] Liu J, Yang L, Tian H, Ma Q. Cathepsin D is involved in the oxygen and glucose deprivation/reperfusion-induced apoptosis of astrocytes. Int J Mol Med. 2016;38:1257–63. 27573911 10.3892/ijmm.2016.2709

[59] Zhang X, Chen C, Liu Y. Navigating the metabolic maze: anomalies in fatty acid and cholesterol processes in Alzheimer’s astrocytes. Alzheimer’s Res Therapy. 2024;16:63. 10.1186/s13195-024-01430-x PMC10960454 38521950

[60] Zeng J, Lo CH, Editorial. Lipid metabolism dysregulation in Obesity-Related diseases and neurodegeneration. Front Endocrinol. 2025;16. 10.3389/fendo.2025.1564003 PMC11850260 40007811

[61] Yang D, Wang X, Zhang L, Fang Y, Zheng Q, Liu X, et al. Lipid metabolism and storage in neuroglia: role in brain development and neurodegenerative diseases. Cell and Bioscience. BioMed Central Ltd; 2022. 10.1186/s13578-022-00828-0 PMC9277953 35831869

[62] Ortiz-Rodriguez A, Arevalo M-A. The contribution of astrocyte autophagy to systemic metabolism. Int J Mol Sci. 2020. 10.3390/ijms21072479 PMC7177973 32260050

[63] Alam S, Afsar SY, Van Echten-Deckert G. S1P released by SGPL1-Deficient astrocytes enhances astrocytic ATP production via S1PR2,4, thus keeping autophagy in check: potential consequences for brain health. Int J Mol Sci. 2023. 10.3390/ijms24054581 PMC10003137 36902011

[64] Sha L, Li J, Shen H, Wang Q, Meng P, Zhang X, et al. LHPP-mediated inorganic pyrophosphate hydrolysis-driven lysosomal acidification in astrocytes regulates adult neurogenesis. Cell Rep. 2023;42:112975. 37573508 10.1016/j.celrep.2023.112975

[65] Zhao N, Liu C-C, Qiao W, Bu G, Apolipoprotein E. Receptors, and modulation of Alzheimer’s disease. Biol Psychiatry. 2018;83:347–57. 28434655 10.1016/j.biopsych.2017.03.003 PMC5599322

[66] Huang Y, Mahley RW, Apolipoprotein E, Pt. 3–12.

[67] Farmer BC, Kluemper J, Johnson LA. Apolipoprotein E4 alters astrocyte fatty acid metabolism and lipid droplet formation. Cells. 2019;8. 10.3390/cells8020182 PMC6406677 30791549

[68] Schmukler E, Solomon S, Simonovitch S, Goldshmit Y, Wolfson E, Michaelson DM, et al. Altered mitochondrial dynamics and function in APOE4-expressing astrocytes. Cell Death Dis. 2020;11:578. 32709881 10.1038/s41419-020-02776-4 PMC7382473

[69] Larramona-Arcas R, González-Arias C, Perea G, Gutiérrez A, Vitorica J, García-Barrera T, et al. Sex-dependent calcium hyperactivity due to lysosomal-related dysfunction in astrocytes from APOE4 versus APOE3 gene targeted replacement mice. Mol Neurodegeneration. 2020;15:35. 10.1186/s13024-020-00382-8 PMC7285605 32517777

[70] Prasad H, Rao R. Amyloid clearance defect in ApoE4 astrocytes is reversed by epigenetic correction of endosomal pH. Proc Natl Acad Sci USA. 2018;115:E6640–9. 29946028 10.1073/pnas.1801612115 PMC6048470

[71] Nuriel T, Peng KY, Ashok A, Dillman AA, Figueroa HY, Apuzzo J et al. The endosomal-lysosomal pathway is dysregulated by APOE4 expression in vivo. Front NeuroSci. 2017;11. 10.3389/fnins.2017.00702 PMC5733017 29311783

[72] Persson T, Lattanzio F, Calvo-Garrido J, Rimondini R, Rubio-Rodrigo M, Sundström E, et al. Apolipoprotein E4 elicits lysosomal cathepsin D release, decreased Thioredoxin-1 levels, and apoptosis. J Alzheimer’s Disease. 2017;56:601–17. 28035917 10.3233/JAD-150738 PMC5271484

[73] Lee H, Cho S, Kim M-J, Park YJ, Cho E, Jo YS et al. ApoE4-dependent lysosomal cholesterol accumulation impairs mitochondrial homeostasis and oxidative phosphorylation in human astrocytes. Cell Rep. 2023;42. 10.1016/j.celrep.2023.113183 37777962

[74] Hammel G, Zivkovic S, Ayazi M, Ren Y. Consequences and mechanisms of Myelin debris uptake and processing by cells in the central nervous system. Cell Immunol. 2022;380:104591. 36030093 10.1016/j.cellimm.2022.104591

[75] Chen K, Garcia Padilla C, Kiselyov K, Kozai TDY. Cell-specific alterations in autophagy-lysosomal activity near the chronically implanted microelectrodes. Biomaterials. 2023;302:122316. 37738741 10.1016/j.biomaterials.2023.122316 PMC10897938

[76] Ponath G, Ramanan S, Mubarak M, Housley W, Lee S, Sahinkaya FR, et al. Myelin phagocytosis by astrocytes after Myelin damage promotes lesion pathology. Brain. 2017;140:399–413. 28007993 10.1093/brain/aww298 PMC5841057

[77] Wang S, Deng J, Fu H, Guo Z, Zhang L, Tang P. Astrocytes directly clear Myelin debris through endocytosis pathways and followed by excessive gliosis after spinal cord injury. Biochem Biophys Res Commun. 2020;525:20–6. 10.1016/j.bbrc.2020.02.069 32070495

[78] Grajchen E, Hendriks JJA, Bogie JFJ. The physiology of foamy phagocytes in multiple sclerosis. Acta Neuropathol Commun. 2018;6:124. 30454040 10.1186/s40478-018-0628-8 PMC6240956

[79] Ortiz-Rodriguez A, Acaz-Fonseca E, Boya P, Arevalo MA, Garcia-Segura LM. Lipotoxic effects of palmitic acid on astrocytes are associated with autophagy impairment. Mol Neurobiol. 2019;56:1665–80. 29916142 10.1007/s12035-018-1183-9

[80] Asimakidou E, Saipuljumri EN, Lo CH, Zeng J. Role of metabolic dysfunction and inflammation along the liver–brain axis in animal models with obesity-induced neurodegeneration. Neural Regeneration Res. 2025;20. 10.4103/NRR.NRR-D-23-01770 PMC11438328 38989938

[81] Zeng J, Lo CH, Cheong LYT. Therapeutic targeting of obesity-induced neuroinflammation and neurodegeneration. Front Endocrinol. 2025;15:1456948. 10.3389/fendo.2024.1456948 PMC11781992 39897964

[82] Thaler JP, Yi C-X, Schur EA, Guyenet SJ, Hwang BH, Dietrich MO, et al. Obesity is associated with hypothalamic injury in rodents and humans. J Clin Investig. 2012;122:153–62. 22201683 10.1172/JCI59660 PMC3248304

[83] Varela L, Kim JG, Fernández-Tussy P, Aryal B, Liu ZW, Fernández-Hernando C, et al. Astrocytic lipid metabolism determines susceptibility to diet-induced obesity. Sci Adv. 2023;7:eabj2814. 10.1126/sciadv.abj2814 PMC11323787 34890239

[84] Pla A, Pascual M, Guerri C. Autophagy constitutes a protective mechanism against ethanol toxicity in mouse astrocytes and neurons. PLoS ONE. 2016;11. 10.1371/journal.pone.0153097 PMC4829237 27070930

[85] Lu K, Zimmermann M, Görg B, Bidmon HJ, Biermann B, Klöcker N, et al. Hepatic encephalopathy is linked to alterations of autophagic flux in astrocytes. EBioMedicine. 2019;48:539–53. 31648987 10.1016/j.ebiom.2019.09.058 PMC6838440

[86] Pascua-Maestro R, Diez-Hermano S, Lillo C, Ganfornina MD, Sanchez D. Protecting cells by protecting their vulnerable lysosomes: identification of a new mechanism for preserving lysosomal functional integrity upon oxidative stress. PLoS Genet. 2017;13:e1006603. 28182653 10.1371/journal.pgen.1006603 PMC5325589

[87] Wang F, Gómez-Sintes R, Boya P. Lysosomal membrane permeabilization and cell death. Traffic. 2018;19:918–31. 30125440 10.1111/tra.12613

[88] Giusti V, Kaur G, Giusto E, Civiero L. Brain clearance of protein aggregates: a close-up on astrocytes. Mol Neurodegeneration. 2024;19:5. 10.1186/s13024-024-00703-1 PMC10790381 38229094

[89] Smethurst P, Risse E, Tyzack GE, Mitchell JS, Taha DM, Chen Y-R, et al. Distinct responses of neurons and astrocytes to TDP-43 proteinopathy in amyotrophic lateral sclerosis. Brain. 2020;143:430–40. 32040555 10.1093/brain/awz419 PMC7009461

[90] Yang Y, Song J-J, Choi YR, Kim S, Seok M-J, Wulansari N et al. Therapeutic functions of astrocytes to treat α-synuclein pathology in Parkinson’s disease. Proceedings of the National Academy of Sciences. 2022;119:e2110746119. 10.1073/pnas.2110746119 PMC9304026 35858361

[91] Kim S, Chun H, Kim Y, Kim Y, Park U, Chu J, et al. Astrocytic autophagy plasticity modulates Aβ clearance and cognitive function in Alzheimer’s disease. Mol Neurodegeneration. 2024;19:55. 10.1186/s13024-024-00740-w PMC11267931 39044253

[92] Chow SK, Yu D, MacDonald CL, Buibas M, Silva GA. Amyloid β-peptide directly induces spontaneous calcium transients, delayed intercellular calcium waves and gliosis in rat cortical astrocytes. ASN Neuro. 2010;2:15–23. 10.1042/AN20090035 PMC2810812 20001968

[93] Stenovec M, Trkov S, Lasič E, Terzieva S, Kreft M, Rodríguez Arellano JJ, et al. Expression of Familial alzheimer disease presenilin 1 gene attenuates vesicle traffic and reduces peptide secretion in cultured astrocytes devoid of pathologic tissue environment. GLIA. 2016;64:317–29. 26462451 10.1002/glia.22931 PMC4707995

[94] St-Pierre M-K, Carrier M, González Ibáñez F, Khakpour M, Wallman M-J, Parent M, et al. Astrocytes display ultrastructural alterations and heterogeneity in the hippocampus of aged APP-PS1 mice and human post-mortem brain samples. J Neuroinflamm. 2023;20:73. 10.1186/s12974-023-02752-7 PMC10015698 36918925

[95] Xiao Q, Yan P, Ma X, Liu H, Perez R, Zhu A, et al. Enhancing astrocytic lysosome biogenesis facilitates Aβ clearance and attenuates amyloid plaque pathogenesis. J Neuroscience: Official J Soc Neurosci. 2014;34:9607–20. 10.1523/JNEUROSCI.3788-13.2014 PMC4099542 25031402

[96] Deng Y, Wang S-Y, Wang Q-G, Xu Z-H, Peng Q, Chen S-Y, et al. AVE 0991 suppresses Astrocyte-Mediated neuroinflammation of Alzheimer’s disease by enhancing autophagy. J Inflamm Res. 2023;16:391–406. 36755969 10.2147/JIR.S392599 PMC9900155

[97] Konstantinidis E, Dakhel A, Beretta C, Erlandsson A. Long-term effects of amyloid-beta deposits in human iPSC-derived astrocytes. Mol Cell Neurosci. 2023;125:103839. 36907531 10.1016/j.mcn.2023.103839

[98] Chen X, Gao R, Song Y, Xu T, Jin L, Zhang W, et al. Astrocytic AT1R deficiency ameliorates Aβ-induced cognitive deficits and synaptotoxicity through β-arrestin2 signaling. Prog Neurobiol. 2023;228:102489. 37355221 10.1016/j.pneurobio.2023.102489

[99] Wirth S, Schlößer A, Beiersdorfer A, Schweizer M, Woo MS, Friese MA et al. Astrocytic uptake of posttranslationally modified amyloid-β leads to endolysosomal system disruption and induction of pro-inflammatory signaling. Glia. 2024;n/a. 10.1002/glia.24539 38629411

[100] Gong C, Bonfili L, Zheng Y, Cecarini V, Cuccioloni M, Angeletti M, et al. Immortalized Alzheimer’s disease astrocytes: characterization of their proteolytic systems. Mol Neurobiol. 2023;60:2787–800. 36729287 10.1007/s12035-023-03231-z PMC10039838

[101] Söllvander S, Nikitidou E, Brolin R, Söderberg L, Sehlin D, Lannfelt L, et al. Accumulation of amyloid-β by astrocytes result in enlarged endosomes and microvesicle-induced apoptosis of neurons. Mol Neurodegeneration. 2016;11:38. 10.1186/s13024-016-0098-z PMC4865996 27176225

[102] Domínguez-Prieto M, Velasco A, Tabernero A, Medina JM. Endocytosis and transcytosis of Amyloid-β peptides by astrocytes: A possible mechanism for Amyloid-β clearance in Alzheimer’s disease. J Alzheimer’s Disease. 2018;65:1109–24. 30103329 10.3233/JAD-180332

[103] Zeng X, Cheung SKK, Shi M, Or PMY, Li Z, Liu JYH, et al. Astrocyte-specific knockout of YKL-40/Chi3l1 reduces Aβ burden and restores memory functions in 5xFAD mice. J Neuroinflamm. 2023;20:290. 10.1186/s12974-023-02970-z PMC10693711 38042775

[104] Hong Y, Liu Y, Zhang G, Wu H, Hou Y. Progesterone suppresses Aβ(42)-induced neuroinflammation by enhancing autophagy in astrocytes. Int Immunopharmacol. 2018;54:336–43. 29197800 10.1016/j.intimp.2017.11.044

[105] Li M-Z, Zheng L-J, Shen J, Li X-Y, Zhang Q, Bai X, et al. SIRT1 facilitates amyloid beta peptide degradation by upregulating lysosome number in primary astrocytes. Neural Regeneration Res. 2018;13:2005–13. 10.4103/1673-5374.239449 PMC6183050 30233076

[106] Rose K, Jepson T, Shukla S, Maya-Romero A, Kampmann M, Xu K et al. Tau fibrils induce nanoscale membrane damage and nucleate cytosolic tau at lysosomes. Proceedings of the National Academy of Sciences. 2024;121:e2315690121. 10.1073/pnas.2315690121 PMC11145263 38781206

[107] Martini-Stoica H, Cole AL, Swartzlander DB, Chen F, Wan Y-W, Bajaj L, et al. TFEB enhances astroglial uptake of extracellular Tau species and reduces Tau spreading. J Exp Med. 2018;215:2355–77. 30108137 10.1084/jem.20172158 PMC6122971

[108] Polito VA, Li H, Martini-Stoica H, Wang B, Yang L, Xu Y, et al. Selective clearance of aberrant Tau proteins and rescue of neurotoxicity by transcription factor EB. EMBO Mol Med. 2014;6:1142–60. 25069841 10.15252/emmm.201303671 PMC4197862

[109] Xu Y, Du S, Marsh JA, Horie K, Sato C, Ballabio A, et al. TFEB regulates lysosomal exocytosis of Tau and its loss of function exacerbates Tau pathology and spreading. Mol Psychiatry. 2021;26:5925–39. 32366951 10.1038/s41380-020-0738-0 PMC7609570

[110] Erustes AG, Stefani FY, Terashima JY, Stilhano RS, Monteforte PT, da Silva Pereira GJ, et al. Overexpression of α-synuclein in an astrocyte cell line promotes autophagy Inhibition and apoptosis. J Neurosci Res. 2018;96:160–71. 28573674 10.1002/jnr.24092

[111] Wang P, Lan G, Xu B, Yu Z, Tian C, Lei X, et al. α-Synuclein-carrying astrocytic extracellular vesicles in Parkinson pathogenesis and diagnosis. Translational Neurodegeneration. 2023;12:40. 37620916 10.1186/s40035-023-00372-y PMC10463943

[112] Cavaliere F, Cerf L, Dehay B, Ramos-Gonzalez P, De Giorgi F, Bourdenx M, et al. In vitro α-synuclein neurotoxicity and spreading among neurons and astrocytes using lewy body extracts from Parkinson disease brains. Neurobiol Dis. 2017;103:101–12. 28411117 10.1016/j.nbd.2017.04.011

[113] Hong B, Ohtake Y, Itokazu T, Yamashita T. Glial senescence enhances α-synuclein pathology owing to its insufficient clearance caused by autophagy dysfunction. Cell Death Discovery. 2024;10:50. 38272865 10.1038/s41420-024-01816-8 PMC10811334

[114] Lee H-J, Suk J-E, Patrick C, Bae E-J, Cho J-H, Rho S, et al. Direct transfer of α-Synuclein from neuron to astroglia causes inflammatory responses in synucleinopathies. J Biol Chem. 2010;285:9262–72. 20071342 10.1074/jbc.M109.081125 PMC2838344

[115] Zhang Y, He X, Wu X, Lei M, Wei Z, Zhang X, et al. Rapamycin upregulates glutamate transporter and IL-6 expression in astrocytes in a mouse model of Parkinson’s disease. Cell Death Dis. 2017;8:e2611. 28182002 10.1038/cddis.2016.491 PMC5386462

[116] Henry AG, Aghamohammadzadeh S, Samaroo H, Chen Y, Mou K, Needle E, et al. Pathogenic LRRK2 mutations, through increased kinase activity, produce enlarged lysosomes with reduced degradative capacity and increase ATP13A2 expression. Hum Mol Genet. 2015;24:6013–28. 26251043 10.1093/hmg/ddv314

[117] Tsunemi T, Ishiguro Y, Yoroisaka A, Valdez C, Miyamoto K, Ishikawa K, et al. Astrocytes protect human dopaminergic neurons from α-Synuclein accumulation and propagation. J Neuroscience: Official J Soc Neurosci. 2020;40:8618–28. 10.1523/JNEUROSCI.0954-20.2020 PMC7643299 33046546

[118] Morrone Parfitt G, Coccia E, Goldman C, Whitney K, Reyes R, Sarrafha L, et al. Disruption of lysosomal proteolysis in astrocytes facilitates midbrain organoid proteostasis failure in an early-onset Parkinson’s disease model. Nat Commun. 2024;15:447. 38200091 10.1038/s41467-024-44732-2 PMC10781970

[119] Pereira GJS, Hirata H, Fimia GM, do Carmo LG, Bincoletto C, Han SW, et al. Nicotinic acid adenine dinucleotide phosphate (NAADP) regulates autophagy in cultured astrocytes. J Biol Chem. 2011;286:27875–81. 21610076 10.1074/jbc.C110.216580 PMC3151033

[120] Vest RT, Chou C-C, Zhang H, Haney MS, Li L, Laqtom NN, et al. Small molecule C381 targets the lysosome to reduce inflammation and ameliorate disease in models of neurodegeneration. Proc Natl Acad Sci USA. 2022;119:e2121609119. 35259016 10.1073/pnas.2121609119 PMC8931323

[121] Chin MY, Ang K-H, Davies J, Alquezar C, Garda VG, Rooney B, et al. Phenotypic screening using High-Content imaging to identify lysosomal pH modulators in a neuronal cell model. ACS Chem Neurosci. 2022;13:1505–16. 35522480 10.1021/acschemneuro.1c00804 PMC9121341

[122] Li B, Liu T, shen Y, Qin J, Chang X, Wu M et al. TFEB/LAMP2 contributes to PM0.2-induced autophagy-lysosome dysfunction and alpha-synuclein dysregulation in astrocytes. J Environ Sci. 2023. 10.1016/j.jes.2023.09.036 38844312

[123] Zeng J, Acin-Perez R, Assali EA, Martin A, Brownstein AJ, Petcherski A, et al. Restoration of lysosomal acidification rescues autophagy and metabolic dysfunction in non-alcoholic fatty liver disease. Nat Commun. 2023;14:2573. 37142604 10.1038/s41467-023-38165-6 PMC10160018

[124] Lo CH, O’Connor LM, Loi GWZ, Saipuljumri EN, Indajang J, Lopes KM, et al. Acidic nanoparticles restore lysosomal acidification and rescue metabolic dysfunction in pancreatic β-Cells under lipotoxic conditions. ACS Nano. 2024;18:15452–67. 38830624 10.1021/acsnano.3c09206 PMC11192035

[125] Trudeau KM, Colby AH, Zeng J, Las G, Feng JH, Grinstaff MW, et al. Lysosome acidification by photoactivated nanoparticles restores autophagy under lipotoxicity. J Cell Biol. 2016;214:25–34. 27377248 10.1083/jcb.201511042 PMC4932370

[126] Dai D, He L, Chen Y, Zhang C. Astrocyte responses to nanomaterials: functional changes, pathological changes and potential applications. Acta Biomater. 2021;122:66–81. 33326883 10.1016/j.actbio.2020.12.013

[127] Lo CH, Zeng J. Defective lysosomal acidification: a new prognostic marker and therapeutic target for neurodegenerative diseases. Translational Neurodegeneration. 2023;12:29. 37287072 10.1186/s40035-023-00362-0 PMC10249214

[128] Quick JD, Silva C, Wong JH, Lim KL, Reynolds R, Barron AM, et al. Lysosomal acidification dysfunction in microglia: an emerging pathogenic mechanism of neuroinflammation and neurodegeneration. J Neuroinflamm. 2023;20:185. 10.1186/s12974-023-02866-y PMC10403868 37543564

[129] Koh J-Y, Kim HN, Hwang JJ, Kim Y-H, Park SE. Lysosomal dysfunction in proteinopathic neurodegenerative disorders: possible therapeutic roles of cAMP and zinc. Mol Brain. 2019;12:18. 30866990 10.1186/s13041-019-0439-2 PMC6417073

[130] Lee H, Koh JY. Roles for H+/K+-ATPase and zinc transporter 3 in cAMP-mediated lysosomal acidification in Bafilomycin A1-treated astrocytes. GLIA. 2021;69:1110–25. 33314298 10.1002/glia.23952

[131] Kim HN, Seo BR, Kim H, Koh JY. Cilostazol restores autophagy flux in Bafilomycin A1-treated, cultured cortical astrocytes through lysosomal reacidification: roles of PKA, zinc and Metallothionein 3. Sci Rep. 2020;10:9175. 32514052 10.1038/s41598-020-66292-3 PMC7280249

[132] Shim MS, Kim KY, Bu JH, Nam HS, Jeong SW, Park TL et al. Elevated intracellular cAMP exacerbates vulnerability to oxidative stress in optic nerve head astrocytes Article. Cell Death Dis. 2018;9. 10.1038/s41419-017-0171-8 PMC5833440 29459737

[133] Ogura M, Taniura H, Nakamichi N, Yoneda Y. Upregulation of the glutamine transporter through transactivation mediated by cAMP/protein kinase A signals toward exacerbation of vulnerability to oxidative stress in rat neocortical astrocytes. J Cell Physiol. 2007;212:375–85. 17323379 10.1002/jcp.21031

[134] Song W, Wang F, Savini M, Ake A, di ronza A, Sardiello M, et al. TFEB regulates lysosomal proteostasis. Hum Mol Genet. 2013;22:1994–2009. 23393155 10.1093/hmg/ddt052

[135] Chandra S, Jana M, Pahan K. Aspirin induces lysosomal biogenesis and attenuates amyloid plaque pathology in a mouse model of Alzheimer’s disease via PPARα. J Neuroscience: Official J Soc Neurosci. 2018;38:6682–99. 10.1523/JNEUROSCI.0054-18.2018 PMC6067079 29967008

[136] Tanaka Y, Suzuki G, Matsuwaki T, Hosokawa M, Serrano G, Beach TG, et al. Progranulin regulates lysosomal function and biogenesis through acidification of lysosomes. Hum Mol Genet. 2017;26:969–88. 28073925 10.1093/hmg/ddx011

[137] Caliò A, Segala D, Munari E, Brunelli M, Martignoni G. MiT family translocation renal cell carcinoma: from the early descriptions to the current knowledge. Cancers. 2019;11. 10.3390/cancers11081110 PMC6721505 31382581

[138] Perera RM, Stoykova S, Nicolay BN, Ross KN, Fitamant J, Boukhali M, et al. Transcriptional control of autophagy-lysosome function drives pancreatic cancer metabolism. Nature. 2015;524:361–5. 26168401 10.1038/nature14587 PMC5086585

[139] Beard E, Lengacher S, Dias S, Magistretti PJ, Finsterwald C. Astrocytes as key regulators of brain energy metabolism: new therapeutic perspectives. Front Physiol. 2021;12:825816. 35087428 10.3389/fphys.2021.825816 PMC8787066

[140] Cisneros J, Belton TB, Shum GC, Molakal CG, Wong YC. Mitochondria-lysosome contact site dynamics and misregulation in neurodegenerative diseases. Trends Neurosci. 2022;45:312–22. 35249745 10.1016/j.tins.2022.01.005 PMC8930467

[141] Lee J-H, Yang D-S, Goulbourne CN, Im E, Stavrides P, Pensalfini A, et al. Faulty autolysosome acidification in Alzheimer’s disease mouse models induces autophagic build-up of Aβ in neurons, yielding senile plaques. Nat Neurosci. 2022;25:688–701. 35654956 10.1038/s41593-022-01084-8 PMC9174056

[142] Sun W, Liu Z, Jiang X, Chen MB, Dong H, Liu J et al. Spatial and single-cell transcriptomics reveal neuron-astrocyte interplay in long-term memory. BioRxiv. 2023;2023.03.20.533566.

[143] Kodam P, Sai Swaroop R, Pradhan SS, Sivaramakrishnan V, Vadrevu R. Integrated multi-omics analysis of Alzheimer’s disease shows molecular signatures associated with disease progression and potential therapeutic targets. Sci Rep. 2023;13:3695. 36879094 10.1038/s41598-023-30892-6 PMC9986671

[144] O’Connor LM, O’Connor BA, Zeng J, Lo CH. Data Mining of Microarray Datasets in Translational Neuroscience. Brain Sciences. 2023. 10.3390/brainsci13091318 PMC10527016 37759919

[145] O’Connor LM, O’Connor BA, Lim S, Bin, Zeng J, Lo CH. Integrative multi-omics and systems bioinformatics in translational neuroscience: A data mining perspective. J Pharm Anal. 2023;13:836–50. 37719197 10.1016/j.jpha.2023.06.011 PMC10499660

[146] Lo CH. Towards clinical translation of biomarkers and therapies targeting autolysosomal acidification dysfunction in neuroinflammation and neurodegeneration—an interview with prof. David Rubinsztein. Aging Pathobiology Ther. 2024;6:138–40.

[147] Rama Rao KV, Kielian T. Astrocytes and lysosomal storage diseases. Neuroscience. 2016;323:195–206. Available from: https://www.sciencedirect.com/science/article/pii/S0306452215005035 10.1016/j.neuroscience.2015.05.061 PMC4664580 26037807

[148] Bosch ME, Kielian T. Astrocytes in juvenile neuronal ceroid lipofuscinosis (CLN3) display metabolic and calcium signaling abnormalities. J Neurochem. 2019;148:612–24. Available from: 10.1111/jnc.14545 10.1111/jnc.14545 PMC7233413 29964296

[149] Platt FM, d’Azzo A, Davidson BL, Neufeld EF, Tifft CJ. Lysosomal storage diseases. Nat Rev Dis Primers. 2018;4:27. Available from: 10.1038/s41572-018-0025-4 10.1038/s41572-018-0025-4 30275469

[150] Lange J, Haslett LJ, Lloyd-Evans E, Pocock JM, Sands MS, Williams BP et al. Compromised astrocyte function and survival negatively impact neurons in infantile neuronal ceroid lipofuscinosis. Acta Neuropathol Commun. 2018;6:74. Available from: 10.1186/s40478-018-0575-4 10.1186/s40478-018-0575-4 PMC6081811 30089511

[151] Liddelow SA, Guttenplan KA, Clarke LE, Bennett FC, Bohlen CJ, Schirmer L et al. Neurotoxic reactive astrocytes are induced by activated microglia. Nature. 2017;541:481–7. Available from: 10.1038/nature21029 10.1038/nature21029 PMC5404890 28099414

[152] Konishi H, Okamoto T, Hara Y, Komine O, Tamada H, Maeda M, et al. Astrocytic phagocytosis is a compensatory mechanism for microglial dysfunction. EMBO J. 2020;39:e104464. 32959911 10.15252/embj.2020104464 PMC7667883

[153] Chung W-S, Clarke LE, Wang GX, Stafford BK, Sher A, Chakraborty C, et al. Astrocytes mediate synapse elimination through MEGF10 and MERTK pathways. Nature. 2013;504:394–400. 24270812 10.1038/nature12776 PMC3969024

[154] Lee J-H, Kim J-Y, Noh S, Lee H, Lee SY, Mun JY, et al. Astrocytes phagocytose adult hippocampal synapses for circuit homeostasis. Nature. 2021;590:612–7. 33361813 10.1038/s41586-020-03060-3

[155] Stevens B, Allen NJ, Vazquez LE, Howell GR, Christopherson KS, Nouri N, et al. The classical complement cascade mediates CNS synapse elimination. Cell. 2007;131:1164–78. 18083105 10.1016/j.cell.2007.10.036

[156] Chu Y, Jin X, Parada I, Pesic A, Stevens B, Barres B et al. Enhanced synaptic connectivity and epilepsy in C1q knockout mice. Proceedings of the National Academy of Sciences. 2010;107:7975–80. 10.1073/pnas.0913449107 PMC2867906 20375278

[157] Schafer DP, Lehrman EK, Kautzman AG, Koyama R, Mardinly AR, Yamasaki R, et al. Microglia sculpt postnatal neural circuits in an activity and Complement-Dependent manner. Neuron. 2012;74:691–705. 22632727 10.1016/j.neuron.2012.03.026 PMC3528177

[158] Deng D, Wang B, Guan Y, Zheng H, Mutlu AS, Wang MC. Quantitative profiling of lysosomal pH heterogeneity using fluorescence lifetime imaging microscopy. BioRxiv. 2023;2023.09.25.559395. 10.1091/mbc.E23-06-0220 PMC11974955 39878653

[159] Prakash P, Jethava KP, Korte N, Izquierdo P, Favuzzi E, Rose IVLL, et al. Monitoring phagocytic uptake of amyloid Β into glial cell lysosomes in real time. Chem Sci. 2021;12:10901–18. 34476070 10.1039/d1sc03486c PMC8372545

[160] Damisah EC, Hill RA, Rai A, Chen F, Rothlin CV, Ghosh S, et al. Astrocytes and microglia play orchestrated roles and respect phagocytic territories during neuronal corpse removal in vivo. Sci Adv. 2020;6:eaba3239. 32637606 10.1126/sciadv.aba3239 PMC7319765

[161] Lian H, Litvinchuk A, Chiang AC-A, Aithmitti N, Jankowsky JL, Zheng H. Astrocyte-Microglia Cross Talk through Complement Activation Modulates Amyloid Pathology in Mouse Models of Alzheimer’s Disease. The Journal of Neuroscience. 2016;36:577 LP – 589. 10.1523/JNEUROSCI.2117-15.2016 PMC4710776 26758846

[162] Rostami J, Holmqvist S, Lindström V, Sigvardson J, Westermark GT, Ingelsson M et al. Human Astrocytes Transfer Aggregated Alpha-Synuclein via Tunneling Nanotubes. The Journal of Neuroscience. 2017;37:11835 LP – 11853. 10.1523/JNEUROSCI.0983-17.2017 PMC5719970 29089438

[163] Scheiblich H, Dansokho C, Mercan D, Schmidt SV, Bousset L, Wischhof L, et al. Microglia jointly degrade fibrillar alpha-synuclein cargo by distribution through tunneling nanotubes. Cell. 2021;184:5089–e510621. 34555357 10.1016/j.cell.2021.09.007 PMC8527836

[164] Wiersma VI, Polymenidou M. Sharing is caring: the benefits of distributing protein aggregates among microglial networks. Neuron. 2021;109:3228–30. 34672979 10.1016/j.neuron.2021.10.008

[165] Gotoh M, Miyamoto Y, Ikeshima-Kataoka H. Astrocytic neuroimmunological roles interacting with microglial cells in neurodegenerative diseases. Int J Mol Sci. 2023. 10.3390/ijms24021599 PMC9865248 36675113

[166] Jha MK, Jo M, Kim JH, Suk K. Microglia-Astrocyte Crosstalk: An Intimate Molecular Conversation. Neuroscientist. 2019. pp. 227–40. 10.1177/1073858418783959 29931997

[167] Chakraborty R, Nonaka T, Hasegawa M, Zurzolo C. Tunnelling nanotubes between neuronal and microglial cells allow bi-directional transfer of α-Synuclein and mitochondria. Cell Death Dis. 2023;14:329. 37202391 10.1038/s41419-023-05835-8 PMC10195781

[168] Stanca S, Rossetti M, Bongioanni P. Astrocytes as Neuroimmunocytes in Alzheimer’s Disease: A Biochemical Tool in the Neuron–Glia Crosstalk along the Pathogenetic Pathways. International Journal of Molecular Sciences. 2023. 10.3390/ijms241813880 PMC10531177 37762184

[169] Wu X, Liu H, Hu Q, Wang J, Zhang S, Cui W, et al. Astrocyte-Derived extracellular vesicular miR-143-3p dampens autophagic degradation of endothelial adhesion molecules and promotes neutrophil transendothelial migration after acute brain injury. Adv Sci. 2024;11:2305339. 10.1002/advs.202305339 PMC10837358 38044319

